# Recommendations for the design of therapeutic trials for neonatal seizures

**DOI:** 10.1038/s41390-018-0242-2

**Published:** 2018-12-24

**Authors:** Janet S. Soul, Ronit Pressler, Marilee Allen, Geraldine Boylan, Heike Rabe, Ron Portman, Pollyanna Hardy, Sarah Zohar, Klaus Romero, Brian Tseng, Varsha Bhatt-Mehta, Cecil Hahn, Scott Denne, Stephane Auvin, Alexander Vinks, John Lantos, Neil Marlow, Jonathan M. Davis, for the International Neonatal Consortium

**Affiliations:** 1 Boston Children’s Hospital & Harvard Medical School, Boston, MA USA; 2 UCL Great Ormond Street Institute of Child Health, London, UK; 3 Johns Hopkins, Baltimore, Maryland USA; 4 INFANT Research Centre & Department of Paediatrics and Child Health, University College Cork, Cork, Ireland; 5 Brighton and Sussex Medical School, Brighton, England; 6 Novartis Pharma Corp, Cambridge, MA USA; 7 University of Birmingham, Birmingham, UK; 8 INSERM, UMRS1138, University Paris V and University Paris VI, Paris, France; 9 Critical Path Institute, Tucson, Arizona USA; 10 C.S.Mott Children’s Hospital, University of Michigan, Ann Arbor, MI USA; 11 Division of Neurology, The Hospital for Sick Children and Department of Pediatrics, University of Toronto, Toronto, Ontario Canada; 12 Riley Children’s Hospital, Indiana University, Indianapolis, Indiana USA; 13 Pediatric Neurology Department & INSERM U1141, APHP, Robert Debré University Hospital, Paris, France; 14 College of Medicine & Cincinnati Children’s Hospital Medical Center, University of Cincinnati, Cincinnati, OH USA; 15 Children’s Mercy Hospital, Kansas City, Missouri USA; 16 UCL Institute for Women’s Health, University College London, London, UK; 17 The Floating Hospital for Children at Tufts Medical Center and the Tufts Clinical and Translational Science Institute, Boston, MA USA; 18 Critical Path Institute (C-Path) International Neonatal Consortium (INC), Tucson Arizona, USA

## Abstract

Although seizures have a higher incidence in neonates than any other age group and are associated with significant mortality and neurodevelopmental disability, treatment is largely guided by physician preference and tradition, due to a lack of data from well-designed clinical trials. There is increasing interest in conducting trials of novel drugs to treat neonatal seizures, but the unique characteristics of this disorder and patient population require special consideration with regard to trial design. The Critical Path Institute formed a global working group of experts and key stakeholders from academia, the pharmaceutical industry, regulatory agencies, neonatal nurse associations, and patient advocacy groups to develop consensus recommendations for design of clinical trials to treat neonatal seizures. The broad expertise and perspectives of this group were invaluable in developing recommendations addressing: (1) use of neonate-specific adaptive trial designs, (2) inclusion/exclusion criteria, (3) stratification and randomization, (4) statistical analysis, (5) safety monitoring, and (6) definitions of important outcomes. The guidelines are based on available literature and expert consensus, pharmacokinetic analyses, ethical considerations, and parental concerns. These recommendations will ultimately facilitate development of a Master Protocol and design of efficient and successful drug trials to improve the treatment and outcome for this highly vulnerable population.

## Introduction

Seizures are one of the most common neurologic emergencies in neonates, arising in ~3/1000 term live births and are associated with significant mortality and neurodevelopmental disability.^1^ In contrast to seizures in older children, most neonatal seizures result from acute symptomatic etiologies rather than epilepsy. The most common etiologies include neonatal encephalopathy caused by hypoxic-ischemia (HIE), focal ischemia affecting one or more vascular territories (stroke), intracranial hemorrhage, infection, cerebral dysgenesis, and metabolic disturbances.^2^ Neonatal seizures are a major challenge for clinicians because of inconspicuous clinical presentation, variable electro-clinical correlation, and poor response to antiseizure drugs (ASDs). Guidelines for their management lack adequate evidence, due to a lack of sufficient randomized controlled trials.^3–6^ Phenobarbital is used worldwide as a first-line medication^7^ even though it is only effective in < 40–60% of neonates,^2,8^ while second-line medications are often midazolam, phenytoin, or levetiracetam.^2,7^ No new ASDs have been evaluated and licensed for use in neonates, resulting in the off-label use of drugs that have been tested only in older children and adults.^9,10^ This practice carries considerable risk to neonates.^11,12^ Drug trials in neonates pose major ethical dilemmas: balancing the potential risks and benefits of research against harm from inadequately studied treatments, leading to potentially effective treatments being withheld for lack of evidence.^12^ Drug development is also hampered by logistical challenges.^13,14^ There is an urgent need for novel drug development programs that incorporate developmental factors, and for prospective, randomized, controlled trials to test the safety and efficacy of new ASDs in neonates.

**Fig. 1:**
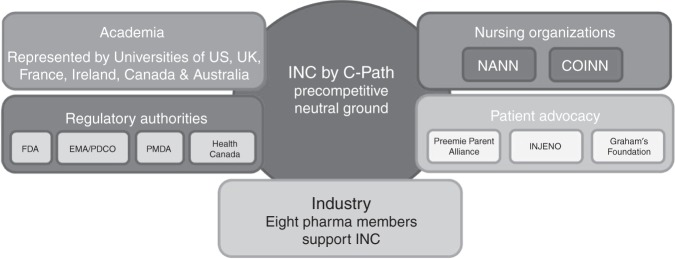
Seizure workgroup collaborators

The International Neonatal Consortium (INC) was formed by the Critical Path Institute (C-Path), an independent, non-profit organization dedicated to accelerating the pace and reducing the costs of medical product development. The seizure working group of INC consists of key stakeholders from research institutions, the pharmaceutical industry, regulatory agencies, nursing groups, patient advocacy, and other organizations to develop guidelines/consensus recommendations for clinical trial design for treatment of neonatal seizures (Fig. 1). The inclusion of broad expertise and perspectives in this international group of experts resulted in a uniquely comprehensive set of recommendations for neonatal seizure trial design.

**Fig. 2:**
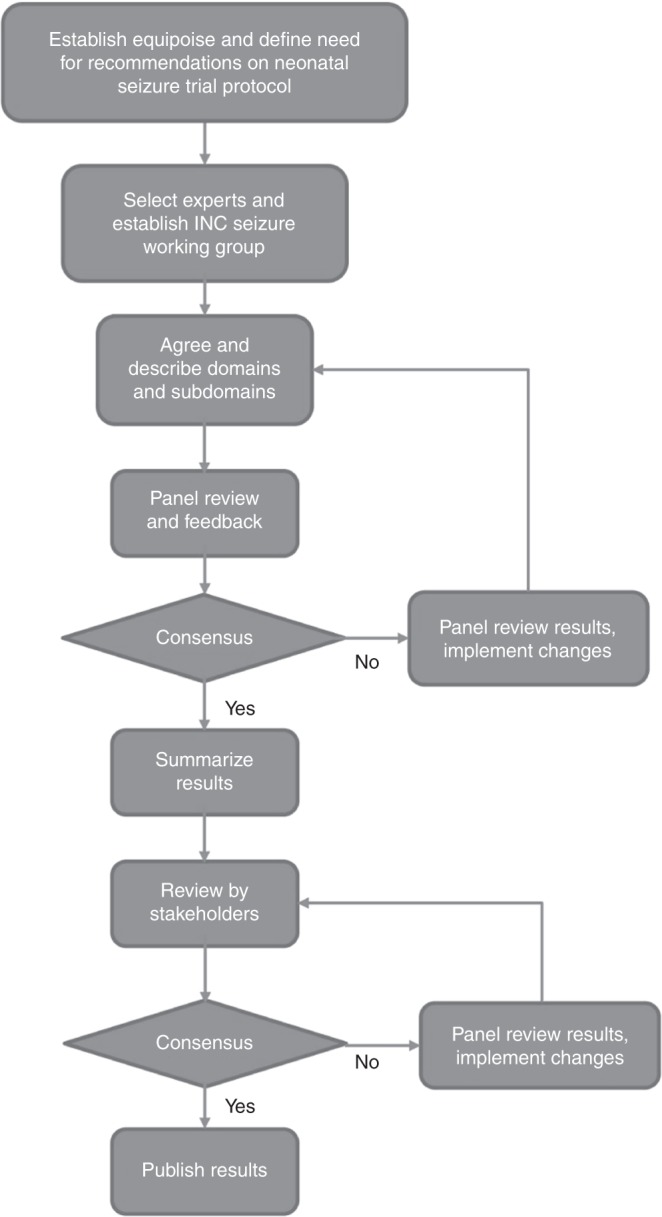
Delphi process to develop neonatal seizure trial recommendations

A modified Delphi process^15^ was applied to develop the recommendations. Key areas for recommendations were agreed. Further topics were added from relevant guidance documents by regulatory authorities. Statements for the recommendations were formulated around the agreed key areas, followed by an iterative process of amendment and agreement until agreement was reached among the working groups before stakeholders provided further feedback and a final consensus was reached (Fig. 2).

This paper describes a synthesis of views expressed by colleagues in EU and North America, which does not necessarily apply to colleagues in other jurisdictions, e.g., some clinicians in Japan thought video-EEG is not feasible for evaluating seizures. Nevertheless, the goal of the stakeholders in writing these recommendations is to ensure more successful and efficient trials of ASDs for this vulnerable population and, consequently, better antiseizure treatments. These recommendations are the crticial first step towards drafting a Master Protocol template to evaluate multiple ASDs or treatments in neonates, which is a priority for many regulatory agencies.^16^

## Protocol design

### Inclusion/exclusion criteria

ASD trials should include neonates with EEG-proven and/or high suspicion of clinical seizures or neonates at high risk of seizures, such as neonates with neonatal encephalopathy caused by HIE, stroke, or intracranial hemorrhage (ICH). Neonates with clinical seizures or high risk of seizures should be included to optimize enrollment and randomization of neonates as early as possible in the course of seizures, but ideally should be randomized only if EEG-proven seizures occur. Trial designs should specify the minimum seizure burden required for enrollment and randomization (see Primary Outcome section), and receipt of pre-approved first-line ASD, if add-on design is used. The following criteria should be considered:

#### Inclusion criteria

A. Age and weight criteria
  1. Postmenstrual age less than 43 + 6/7 weeks, as older infants have different seizure etiologies, seizure pathogenesis, and hepatic/renal function, potentially affecting drug efficacy, safety, and/or drug metabolism and elimination.
  2. Preterm neonates should only be included in the following circumstances:
    - Preterm neonates < 35 weeks’ gestation for Phase 1/2 studies only if sufficient safety and pharmacokinetic data (PK) data available in term neonates.
    - Preterm neonates < 35 weeks’ gestation in Phase 3 only if safety and PK data available for preterm neonates.
    - No absolute birth weight or weight at trial entry minimum required, although PK/PD of drug may require a minimum weight.
B. Etiologies of neonatal seizures: Etiologies of neonatal seizures include moderate to severe HIE, stroke, ICH, infection, cerebral malformations, genetic causes, epilepsy syndromes, and other more rare causes.^2,17^ Etiologies to include in trials should be carefully considered based on anticipated drug mode of action, as some drugs may be indicated or contraindicated for specific genetic etiologies (e.g., KCNQ2/3, SCN2A). Additionally, systemic illness (e.g., severe hepatic or renal dysfunction) and treatments (e.g., hypothermia) associated with specific etiologies could affect drug efficacy, PK, or safety parameters significantly. Inclusion of single vs. multiple etiologies will have substantial effects on trial feasibility and time to completion, and consideration of endpoints, such as PK, safety, and/or efficacy.
  1. All etiologies (except metabolic etiologies) may be appropriate to include in Phase 1/2 trials if PK and safety are main endpoints. Inclusion of all etiologies in early phase trials has the advantage of uncovering exceptional efficacy or safety outcomes for particular etiologies.
  2. Single etiology (e.g., HIE, or hemorrhagic/ischemic etiology) trials may be the best choice for Phase 3 trials testing drug efficacy because subject homogeneity with regard to seizure pathogenesis decreases confounders when comparing treatment arms, although anticipated variability in seizure burden and outcome (even for HIE-only) will still need to be incorporated into the trial design. Conversely, a Phase 3 trial aimed to test a drug’s effectiveness for all neonatal seizures may include multiple etiologies.

#### Exclusion criteria

A. Excluded etiologies
  1. Single etiology trial (e.g., HIE): other causes of seizures such as known brain malformation, genetic disorder, and major congenital malformation.
  2. Multiple etiologies trial: both acute metabolic and inborn errors of metabolism. Metabolic etiologies should be excluded as seizure treatment is largely directed at correcting the metabolic abnormality, whether acute transient metabolic abnormality (e.g., abnormal calcium, glucose, etc.) or inborn error of metabolism, rather than ASDs. Subjects may need to be excluded after enrollment, since etiology often cannot be confirmed at time of seizure onset.
B. ASD(s) already given (other than sedative drugs), per specific trial design.
C. Renal failure: Defined as anuria in the first 24 h after birth, for ASDs with largely renal excretion.
D. Hepatic failure. Hepatic dysfunction requires caution if the ASD under study is predominantly metabolized by the liver
E. Drug clearance safety considerations; organ toxicity contingent on drug clearance profile relative to ontogeny of this unique population.
F. Subjects in whom death seems imminent (hours to several days), as assessed by the treating neonatologist, as early death may preclude assessment of drug efficacy, safety, and/or pharmacokinetics.
G. Participation in other interventional trials, with specific exceptions.^18^
H. Unlikely that parent/neonate can complete evaluation of all follow-up trial endpoints
I. Previous, current, or planned treatment with specific drug(s), based on specific interaction with ASD being tested.

### Choice of comparator

The choice of a comparator for use in neonatal seizure treatment trials is constrained by limitations related to ethical issues, feasibility, and available efficacy and safety data. There is a strong consensus among clinicians, researchers, and parents that it is ethically unacceptable to randomize neonates with seizures to placebo-only treatment. Among the reasons cited were evidence suggesting harm of ineffective treatment of neonatal seizures and some evidence indicating (although not definitively establishing) efficacy of currently used drugs.^8,19,20^ There is also consensus that it would be unethical to delay starting antiseizure treatment in a trial (i.e., no drug for several hours). Placebos could be used to evaluate a new ASD using an “add-on” method, keeping the subjects on identical maintenance treatments with the standard drug (e.g., phenobarbital), then adding a new ASD to one arm and placebo to the other.

Phenobarbital is recommended as the comparator that should be used in trials of new drugs for the following reasons:

A. Phenobarbital is the most common, standard drug used to treat neonatal seizures as a first-line drug in > 90% of tertiary centers.^21^
B. There are relatively few drug interactions for phenobarbital (except induction of hepatic cytochrome affecting metabolism) compared with drugs such as phenytoin or lidocaine.^22–26^
C. Available data suggesting (but not definitively establishing) the efficacy of phenobarbital, although the one published randomized trial was not designed to test superiority and was limited by the crossover design.^8^
D. Phenobarbital has well-documented pharmacokinetics with consistent absorption and elimination compared with other drugs.^22,23,27^
E. There are no controlled data available with alternative ASDs.

Limitations in using phenobarbital as a comparator include lack of definitive data for efficacy of phenobarbital and concerns that high doses of phenobarbital could induce neuronal apoptosis in the neonatal brain.^28^ Phenobarbital should be used as a comparator to assess efficacy or pharmacodynamics, although phenobarbital may not remain the comparator of choice if a new drug is shown to be superior, or at least equivalent, to phenobarbital.

### Treatment arms

The choice of the number and type of treatment arms should be guided primarily by the goals of the trial, keeping the following points in mind.

1. Preliminary trials for testing PK may use one treatment arm, as a control group may not be needed, but most other trials must include a control arm.
2. Larger Phase 2 or 3 trials testing safety and efficacy, including trials with dose-finding design, require a comparison group and stopping rules for an unacceptable safety profile. A control group allows attribution of adverse events to drug vs. underlying disease, given the typical high incidence of adverse events in critically ill neonates with seizures. Similarly, the safety profile of previously used drugs may be known, but new therapies (e.g., hypothermia, new non-seizure drugs) could affect the safety profile of old and new ASDs alike. Thus, trials of previously used ASDs may require a control group to test the safety of these drugs combined with newer therapies.
3. Phase 3 trials to establish efficacy for neonatal seizures will require a comparison group in a superiority designed trial as a non-inferiority trial design is not possible, for the following reasons. A superiority trial design is currently required for neonatal drug approval by the FDA. Superiority does not require first establishing that phenobarbital is efficacious, if phenobarbital is considered “standard” therapy. In principle, efficacy may be demonstrated by superiority or non-inferiority trial designs; however, non-inferiority designs are not currently an option for drug approval in neonates because there is a lack of data from multiple neonatal clinical trials (or placebo-controlled trials of phenobarbital) required to set the margin of a non-inferiority trial. Furthermore, a non-inferiority design would require a larger sample size and phenobarbital would first need to be shown to be effective.^29,30^ For FDA approval, it is not acceptable to conclude similar efficacy with superior safety. For EMA approval, a positive benefit to risk ratio should be demonstrated for Marketing Authorization. Randomized comparative trials are recommended for approval. However, recognizing the feasibility challenges of drug trials for neonatal seizures, the use of a historical control group might be acceptable for approval, provided there is pre-defined matching by age, condition, standard of care, and diagnostic methods.
4. The use of three or more treatment arms is possible if two or more drugs are being compared to a control. In this case, an adaptive design to favor the safest and most effective drug may be preferred.

### Trial design

There are very few trial designs specifically developed for neonates and even fewer for neonatal seizures. Neonate-specific trial designs are being developed following the recent update of ICH E11(R1) guideline in 2016, in which regulatory agencies emphasized the need for extrapolation, simulation, and modeling, and for innovative methodology in pediatric clinical trials.^31^ For example, a unified approach for extrapolation and bridging information was proposed for early phase pediatric studies using different types of data sources to plan early phase clinical trials.^32^ All available information should be used to inform design of neonatal seizure trials, including data available from off-label use of ASDs and expert opinion, to optimize design for small sample trials. All assumptions should be clearly stated, as emphasized by the ICH E11(R1) guideline.^31^

In response to a call by the EU, three multidisciplinary groups are developing dedicated methods for small sample trials (all phases) under frequentist or Bayesian inference,^33^ which could be modified and used for neonatal seizures. Bayesian inference-based designs are well suited to neonatal ASD trials, as previous/current data and expert opinion can be summarized in the prior distribution and used for data analysis. Such innovative methodology with adaptive design was applied in the NEMO dose-finding trial of bumetanide, which included joint modeling of short-term safety and efficacy, but not long-term toxicities.^34^ As the trial was stopped early for serious adverse effects without apparent evidence of efficacy, a novel design was developed for a subsequent early phase trial of levetiracetam (NCT02229123).^35^ Data from these ongoing trials (e.g., Boston bumetanide trial and levetiracetam trials) will need to be incorporated into the design of future neonatal ASD trials.

#### Endpoints

Endpoints must be clearly defined, including endpoints for PK, safety, and/or efficacy, depending on the trial phase (early or late). Recommendations for primary and secondary outcomes to measure drug efficacy and safety are described in the sections on Neonatal Seizure Outcome and Neurologic & Long-term Outcome, and PK measurements in the section on Drug Related Issues. A trial may employ a single endpoint, or two or more endpoints, particularly for Phase 2 & 3 trials (either dual or composite endpoints).

Dual endpoints may be employed in early phase trials, i.e., two independent endpoints used together for modeling and analysis. Clinically meaningful composite endpoints that are expected to act in the same direction (e.g., seizure reduction and maintenance of seizure freedom) may be employed to maximize efficiency of drug testing. For composite endpoints, each endpoint should represent a clinically meaningful efficacy outcome of equal importance, as for seizure reduction and maintenance of seizure freedom. Composite endpoints may not be suitable for early phase trials designed to test PK and safety, as ASD safety and PK parameters are different types of endpoints with unequal importance.

Choice of trial design

The trial design selected should use estimation methods (for early phase exploratory trials) and provide the best evidence (for Phase 2/3 randomized confirmatory trials) with the fewest number of neonates to maximize potential benefit and minimize risk. As such, adaptive trial designs integrating an interim analysis for safety and/or efficacy evaluation are increasingly accepted designs that may provide these advantages.^36^

A. Adaptive design
  Advantages of sequential/adaptive design:
  1. Optimizes the number of included or randomized subjects, which is important given the relatively limited population of eligible neonates with seizures.
  2. Maximizes the number of subjects included or randomized to the most/more effective treatment, since data analyzed sequentially during the trial benefit subsequently enrolled subjects (e.g., prioritize treatments based on optimal seizure reduction).
  3. Trial stops as soon as pre-defined efficacy endpoints have been adequately evaluated, e.g., reaching a pre-defined threshold of increased probability of seizure reduction.
  4. Can adapt one or more design variables, such as treatment group assignment, sample size, dose selection, or even endpoint definition (based on interim estimate of treatment effect), e.g., de-prioritize treatments that miss the pre-defined threshold for seizure reduction probability.
  Requirements to be specified before using an adaptive design:
  An adaptive design requires generation of initial assumptions based on all available data from published trials and observational data. In addition, the stopping rules for these designs depend on the definition and selection of the study endpoints. This limitation regarding endpoints exists in part because clinically meaningful reduction in neonatal seizure burden as a primary endpoint has been defined partly by expert consensus rather than direct evidence of any benefit(s) of seizure reduction. In particular, the large variability in seizure burden in neonates may make an adaptive design based on endpoints or treatment assignment more prone to error with small sample size. A limited sample size in an adaptive design trial may also be too small to adequately test some outcomes completely (e.g., safety and secondary outcomes). Thus, early phase dose escalation studies might need to stipulate a minimum number of subjects prior to escalation if there are limited safety data available for the drug being tested, which favors inclusion of a control arm. Confirmatory randomized trials that use a frequentist approach require a priori consensus on the null hypothesis for superiority testing (what constitutes adequate evidence of a drug effect), which defines the upper and lower bounds for the stopping rules.^37–39^ For confirmatory trials using a Bayesian approach, consensus is needed for the definition of the threshold of the posterior probabilities for efficacy parameters, such as a given increase in the probability of neonatal seizure reduction.
B. Conventional (i.e., non-adaptive) trial design:
  Conventional design may be appropriate for exploratory, early phase trials if there is significant uncertainty regarding many of the initial assumptions, which limits generation of critical aspects of an adaptive trial design.
C. Crossover design:
  Crossover design is discouraged because it requires a significantly larger number of subjects to test most outcomes (efficacy, safety, PK/PD), and because there is limited interpretation of all outcomes if majority of subjects crossover, as is likely given high failure rate of current drugs for neonatal seizures.

#### Randomization

A central, pre-defined randomization strategy should be employed for all Phase 2B or Phase 3 trials with two or more treatment arms, taking into account stratification considerations to balance treatment arms (see below). Randomization should occur as soon as possible after seizure onset, and within 24 h of seizure onset, to ensure that the study drug is tested prior to natural resolution of seizures. Randomization assignment should also take place as close as possible to the time of study drug administration, as some stratification covariates (e.g., hypothermia, severity of encephalopathy) may change between time of enrollment and randomization.

Balancing treatment arms:

The randomization method should ensure balance across only essential subject characteristics that can affect one or more outcomes and that can be readily identified at enrollment. The method of randomization (e.g., stratified block randomization or minimization) should be considered according to the number of variables selected. Subject characteristics to consider:

A. Seizure etiology and seizure severity would ideally be balanced between treatment arms but are often impossible to determine at the time of enrollment or randomization. Instead, these can be pre-defined subgroups, which are adjusted for at the analysis stage (see Statistical Analysis Plan). This may be particularly important for dose-finding studies where the optimal dose may vary with seizure severity.
B. Centers should be balanced whenever possible in multicenter studies to account for center differences in populations and neonatal and neurological management.
C. Therapeutic hypothermia (TH) treatment may affect drug PK, safety, and efficacy, and is usually known at the time of enrollment and randomization. It is important to balance the treatment arms with regard to TH, due to limited numbers of neonates with HIE not treated with TH.
D. Gestational age (GA) at birth is not essential because of the lesser effect on outcomes compared with the characteristics listed above (GA at birth can be pre-defined and adjusted for at the analysis stage, see Statistical Analysis Plan).

#### Masking (blinding)

Investigators, treating clinicians, and subjects’ family members should all be masked to treatment assignment for randomized trials with more than one treatment arm to preserve integrity of trial conduct, data collection, and data analysis. This is particularly important for trials of ASDs, since natural variation in seizure burden over time could be falsely attributed to treatment success or failure. The Data and Safety Monitoring Board (DSMB) should be masked for the same reason, although could be unmasked early if there are safety concerns or the possibility of achieving a pre-defined endpoint prior to study completion.

#### Safety

Neonatal seizure treatment trials require safety monitoring by an external DSMB with appropriate expertise, including pediatric neurologists/neurophysiologists and neonatologists with expertise in neonatal seizures and the seizure etiologies being included in the trial, and a statistician with trial experience. This is particularly important since adverse events of many kinds (neurologic and non-neurologic) occur commonly in critically ill neonates with seizures caused by asphyxia/HIE or other systemic disorders (including serious adverse events such as death). The frequent occurrence of adverse events means that a control arm is essential to compare rates of adverse events for all but certain early phase trials of drug PK. Adverse events and estimated rates of those events should be defined prior to trial commencement, including events and laboratory values related to the underlying condition(s) and study drug, given the possibility of the study drug resulting in increased (rather than reduced) seizure burden. The rate of adverse events may vary according to the population of neonates being studied, seizure etiology, and study drug toxicities, so needs to be defined for each trial. Stopping rules for safety outcomes should be separate from efficacy or other primary outcomes, including for dose-finding trials. The investigators and DSMB should monitor adverse events closely throughout the neonatal hospitalization. Serious adverse events such as death and illnesses/injury leading to permanent disability should be reviewed and reported immediately to the DSMB and governing IRB(s), according to the definitions set out by the FDA/EMA/NIH.^40,41^ Finally, long-term outcome should be included as both a safety and efficacy measure (see Section on Neurologic and Long-term Outcome Measures).

#### Statistical plan, stopping rules and analyses

A. Sample size considerations
  The statistical approach should take into account the sample size needed to evaluate primary and secondary outcomes adequately, and employ pre-defined endpoints and analysis approach for all outcomes. If a frequentist analysis plan is chosen, a clear definition and justification of the null and alternative hypothesis should be specified (e.g., H0: lack of difference in the proportion of controlled seizures between treatment and control groups). Moreover, the power and alpha parameters choices should be clearly identified at trial initiation, typically, 1-β = 0.8, *α* = 0.05. Any increase of alpha above 5% or decrease of power value below 80% should be justified. If a Bayesian analysis plan is chosen, prior distribution of the estimated parameters should be clearly identified (e.g., expected probability of seizure reduction for given treatment). The construction and the prior distribution choice of parameters should be explained and justified. A decision rule on how to categorize a treatment win should also be pre-specified (e.g., targeted increase in the probability of seizure reduction, given an intervention), using expert opinion, trial data, or any other source of available data.
  Sample size for efficacy trials should be defined to detect a clinically meaningful reduction in seizure burden (see Section on Neonatal Seizure Outcome Measures), keeping the follow points in mind:
  1. Outcome measures likely exert the greatest influence on sample size.^42^
  2. Large variability in seizure burden in subjects affects sample size significantly and may not be accounted for by randomization alone.
  3. Given the challenges of enrolling sufficient numbers of eligible neonates with seizures, the feasibility of the proposed sample size needs to be considered with regard to available subjects, cost, and time to study completion.
B. Stopping rules
  1. The plan for any interim analyses should be specified in advance (e.g., interim analyses at given sample size targets), together with stopping guidelines (e.g., stopping at a given increase in the probability of seizure control). Even planned interim analyses may require an increase in sample size, which should be detailed in the original design and statistical analysis plan, as possible.
  2. Separate stopping rules are needed for safety and efficacy.
    - Stopping rules for safety should include continuous interim analysis for safety monitoring based on previously defined distribution (not rate) of adverse events in the enrolled population and according to the predicted potential toxicities of the study drug specific to neonates with the included seizure etiologies.
    - Stopping rules for efficacy should be defined by clinical investigators and the trial statistician (e.g., reaching a pre-defined increase in the probability of seizure reduction). However, it should be difficult to stop a trial early for efficacy, due to the need to analyze long-term assessments of safety and neurodevelopmental outcome.
  3. Rare reasons for late or substantial changes to the trial design or analysis plan could include major errors in initial statistical assumptions, e.g., differences in data distribution or model of seizure reduction. This should be avoided whenever possible and will require substantial changes to the statistical analysis plan.
C. Statistical analysis plan
  There are several issues specific to drug trials for neonatal seizures that should be addressed in planning the statistical analyses.
  1. Severity of seizure burden is one of the most important covariates (often the most important) that must be considered in any analysis of drug response/efficacy. Currently, there is no universally accepted definition of severity of seizure burden, which varies with seizure etiology and patient population. Since seizure severity currently cannot be determined reliably at time of randomization, subjects cannot be stratified by seizure severity. The statistical analysis plan will therefore require modeling that includes subgroup analysis by seizure severity at trial completion, as a pre-planned post-hoc analysis for any early or late phase trials testing dose response or efficacy.
  2. Seizure etiologies also may not be balanced among treatment groups if more than one etiology is included, since seizure etiology is often unknown at time of enrollment or randomization. Instead, trial designs should include a pre-planned post-hoc analysis to determine whether treatment effect differs for different seizure etiologies. Adjusting for these types of characteristics in the analysis improves the precision of the overall treatment effect (i.e., provides a smaller standard error).
  3. There are other potential clinical covariates that may affect measurement of the efficacy, safety, or PK of ASDs in neonates, such as GA at birth, postmenstrual age at randomization, weight, or factors specific to the drug (e.g., renal or hepatic clearance), which should be accounted for in the analysis plan.

## Neonatal seizure outome measures

### Introduction

Continuous video electroencephalography (cvEEG) is the gold standard for seizure detection in neonates,^43^ as it is well established that the clinical evaluation of seizures without cvEEG confirmation can lead to both over- and under-diagnosis.^44^ Continuous video-EEG monitoring is essential since subclinical seizures occur in most neonates and as many as 16% have only subclinical seizures,^2^ particularly those with severe encephalopathy and/or receiving sedative medications. Both the FDA and EMA recognize the incontrovertible data showing the superiority of multichannel cvEEG monitoring for accurate detection of neonatal seizures compared with reduced montage devices such as aEEG, or EEG without video. Seizures are accurately detected with high inter-rater reliability when read by pediatric neurophysiologists, who should be the readers for any ASD trial evaluating drug response or efficacy.^45^ Thus, multichannel (minimum 8-20 channels) cvEEG monitoring is needed for accurate detection of seizures to determine drug response in neonatal seizure trials in order to obtain regulatory approval from the FDA and EMA. The measure of choice to assess drug efficacy is neonatal seizure burden measured in minutes of seizure activity per hour by cvEEG.^8,34,46,47^

To establish a robust primary outcome measure for a clinical trial of neonatal seizure treatment, we reviewed the available literature regarding treatment of neonatal seizures and performed an international survey of neonatologists, pediatric neurologists, and other expert clinicians who treat neonatal seizures. The literature review showed that a pre-treatment baseline period and response to treatment were rarely defined clearly, although an 80% reduction in seizure burden was the most commonly cited desired drug response. Although the international survey yielded variable opinions regarding the pre-treatment seizure burden that should prompt treatment, an 80% reduction in seizure burden was designated an optimal drug response. See “Supplemental Appendix 1 *(online)*” for Literature Review and Results of International Survey. As there is no current consensus on how treatment efficacy for neonatal seizures should be measured or data that define what specific reduction in seizure burden improves neurologic outcome, we propose the following:

### Proposed neonatal seizure outcome measure

#### Seizure burden prior to treatment—the baseline period

**Fig. 3:**
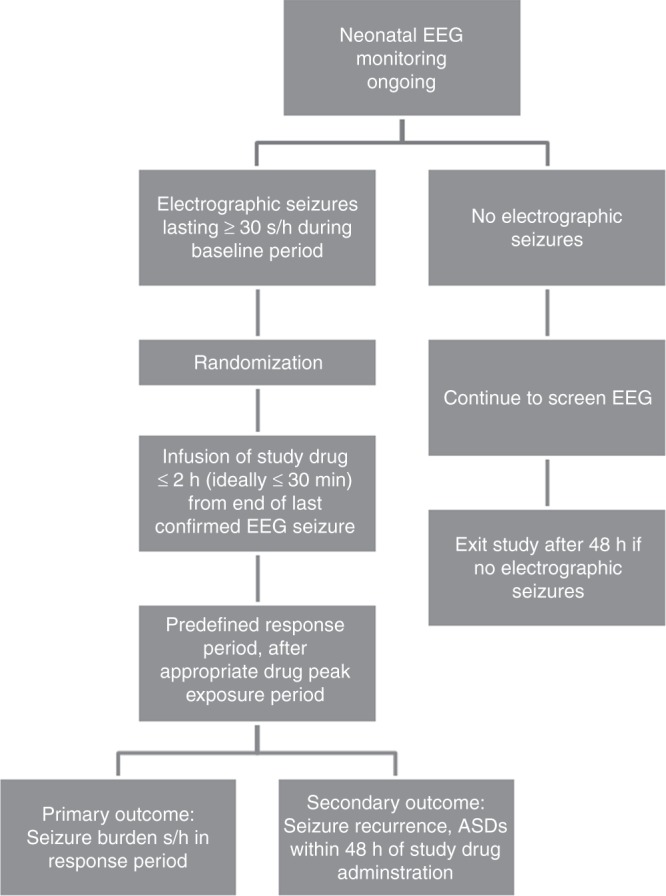
Seizure treatment diagram

A baseline period is needed to establish entry criteria to a trial. A cumulative electrographic seizure burden of at least 30 seconds/hour (sec/h) during the baseline period is needed for a subject to be eligible for randomization. Confirmation of electrographic seizures by a local or central neonatal EEG expert is required. Study drug administration should start as soon as possible after the 30 sec/h of seizure activity is confirmed by the expert, ideally within 30 min (maximum two hours) of the end of the last confirmed electrographic seizure, or with ongoing confirmed electrographic seizures. If study drug cannot be administered within two hours of the last seizure, then study drug should not be administered unless and until another 30 sec/h of seizure activity occurs. This is necessary to avoid randomizing neonates whose seizures have resolved spontaneously before administration of study drug. Seizure Treatment Diagram outlines an example of a trial protocol (Figure 3).

#### The primary seizure outcome measure

The primary outcome measure in a late phase drug efficacy trial should be the total seizure burden (in minutes/hour) per subject in the response period (i.e., continuous outcome measure) after study drug administration. A period of ~30 min following drug administration is required for peak drug exposure, a time period which may need to be reduced or extended depending on the drug’s properties. The response period should be defined as a ≥ 2 h period following the time of expected peak drug exposure. The comparison of seizure burden/subject between treatment arms may need to be adjusted for overall seizure burden, particularly if the severity of seizure burden is not balanced among treatment groups (e.g., by chance). The primary outcome may also be measured as the reduction in seizure burden during the response period, compared with a ≤ 2 h baseline period immediately prior to study drug administration, if the baseline period is of adequate duration in sufficient numbers of subjects. An adequate baseline period is more likely to be available in trials of second-line drugs or add-on therapies.

For early phase dose-finding trials, a responder rate needs to be defined as a specific, clinically meaningful reduction in seizure burden. In early phase trials, reduction in seizure burden during a response period may be compared with the baseline period, but will need to be adjusted for total seizure burden, given the typically small sample size in early phase trials. For example, a primary outcome could be a > 30% reduction in seizure burden in neonates receiving study drug compared with control neonates receiving standard treatment. A smaller reduction in seizure burden may be considered appropriate for neonates with severe seizures, such as status epilepticus.

Efficacy of the study drug (or meaningful drug response for early phase trials), should be demonstrated by a meaningful reduction in seizure burden in the study drug group that is statistically and clinically superior to that of the control comparator group. Notably, the baseline period may be very short for some subjects, and will likely have variable duration for all subjects in a trial. Use of a control arm can mitigate the effect of variable baseline periods and fluctuating seizure burden, and overall seizure burden can be used as a covariate in the analysis of drug response or efficacy. Drug response or efficacy should also be evaluated by the relationship between seizure reduction and dose exposure, determined by measured drug levels and population PK analysis.

Seizure recurrence is another outcome measure that may be used as an equally important outcome measure, i.e., combined with a reduction in seizure burden as a composite endpoint for a primary outcome, or as a secondary outcome measure. The recurrence of seizures is measured by total seizure burden from the end of the defined response period until 48 h after study drug administration (i.e., 48 h for acute seizures from ischemic/hemorrhagic etiologies, up to 72 h for other etiologies, e.g., infection, genetic/malformation). This is contingent on maintenance of therapeutic levels of study drug (Fig. 3 Seizure Treatment Diagram).^48^ Thus, cvEEG monitoring should be continued for at least 48 h after study drug administration and at least 24 h after the last confirmed EEG seizure.

Finally, administration of rescue ASDs could be used as another outcome measure of drug response or efficacy, depending on whether or not the trial protocol stipulates when and what doses of ASDs can be administered by the treating clinicians. Administration of rescue ASDs is clearly a less reliable outcome measure if left to the discretion of treating clinicians than if rescue ASDs are stipulated by the trial protocol. As for seizure recurrence as an outcome measure, administration of rescue ASDs should be reported only if there is maintenance of therapeutic levels of study drug. Figure 3 is an example which would need to be adapted to a given study drug and protocol.

## Neurologic and long-term outcome measures

Although the primary efficacy outcome for trials of ASDs in neonates is reduction of seizure burden, any medication that treats seizures may also affect brain development and injury, and risk of later epilepsy. Ideally, an effective and safe ASD would reduce the risk of later epilepsy and neurologic disability. Seizures impair brain function acutely and may exacerbate brain injury,^19^ so there may be a significant benefit to reducing seizure burden. Some ASDs may reduce the incidence or severity of later epilepsy, or have additional neuroprotective effects on the brain which could improve long-term outcome.^49–53^ Thus, demonstration of improved long-term neurologic outcome, including reduced incidence of epilepsy, could be a secondary outcome measure of drug efficacy. Improved long-term neurologic outcome should be a secondary (rather than primary) outcome measure, largely because outcome is primarily determined by seizure etiology, such as the severity and location of any neonatal brain injury, brain malformation, or neurogenetic disorder. Thus any measure of long-term neurologic outcome needs to be correlated first with seizure etiology, including imaging abnormalities.

Conversely, medications used in the neonatal period have the potential to disrupt or adversely influence development of the brain (e.g., via neuronal apoptosis) and other organs, which may be dose dependent.^52,53^ Long-term safety is always an important issue, but the dilemma of “How long is enough?” is a central issue in all neonatal drug trials. For safety concerns, neonates involved in drug trials should be closely monitored throughout the neonatal period (i.e., to 30 days), as well as into infancy and childhood, because some adverse (or beneficial) effects may not be detectable for years. Long-term follow up prolongs trial duration and is expensive as it requires tracking, retention strategies, and assessments of neurodevelopmental outcome. Development is significantly influenced by the child’s environment, which is a confounding variable. Sociodemographic data should be collected at each time point. Nevertheless, the neonatology literature has proven the importance of determining adverse long-term outcomes of neonatal interventions.^54,55^ The lists of secondary outcome measures below are organized by age of assessment and the category of neurologic function or general health being measured. Many of these measures are specific to particular countries or languages, so need to be adapted to local validated tools (complicating comparison in multinational trials). Nevertheless, a comprehensive assessment of neurodevelopmental outcome, later seizures/epilepsy, and general health is needed to determine any beneficial or detrimental effect of ASDs tested in neonates.

### Neonates

Neonatal clinical assessment: The best measures of nervous system function involve neurologic examination and/or observation of a neonate’s behavior. Three standardized exams and one standardized movement assessment (which all require specific training) have been used to assess neurologic integrity, sensory responses, and behavior in full-term neonates with brain injury. Additional measures of brain function and structure that may be affected by ASDs can be assessed prior to hospital discharge, shown in Table 1.

**Table 1:** Secondary outcome measures in the neonate

Criteria	Description
Oral feeding ability	Sufficient infant bottle or breast feeding for growth, partial or total gavage feedings, gastrostomy feedings, or intravenous nutrition required
Health	Respiratory requirements, growth parameters especially head circumference, medications required
Hearing	Neonatal hearing screening tests: 1. Auditory brainstem responses 2. Otoacoustic emissions
Vision	Visual responses to stimuli
Neuroimaging	Brain structural MR imaging with a central reader is preferred; its value as an outcome depends on the homogeneity of conditions defined for the trial, and ideally is obtained at approximately term age before NICU discharge (or by 44 weeks postmenstrual age)
Neonatal clinical assessment^a^	Four assessments are widely used in full-term neonates with brain injury: 1. Amiel-Tison Neurological Assessment at Term^b^ (ATNAT)^70,71^: An exam with 4 outcome categories: Normal; Minor, Moderate or Severe neurological abnormalities/CNS depression 2. Qualitative Assessments of General Movements (GMs): scoring based on observation of a neonate’s spontaneous movements^72,73^ 3. The Hammersmith Infant Neurological Exam^b^ (HINE):^74,75^ an exam that is scored based on calculation of an optimality score 4. NICU Network Neurobehavioral Scale (NNNS):^76^ an exam that generates raw scores on 115 items converted to 13 summary scores

^a^All require training to establish inter-rater reliability

^b^Measures neurologic integrity and sensory responses and behavior that involve neurologic examination and observation of infant behavior

### Toddlers

For safety, adequate assessment of neurologic function requires follow up for at least 18 months to two years, the earliest age at which major neurodevelopmental disability can be reliably determined. Cognition can be assessed more precisely at ≥ 3 years when cognitive and particularly language skills are more complex (Table 2).

**Table 2:** Secondary outcome measures of toddlers

Criteria	Description
Post-neonatal onset epilepsy	Specify seizure type according to ILAE^77,78^
Health	1. Hospitalizations and/or surgeries 2. Medical visits; therapies, other allied professional support, and medications 3. Growth percentiles for height, weight, and head circumference 4. Cardiorespiratory, e.g., limited exercise tolerance, need for respiratory support 5. Gastrointestinal, e.g., need for a special diet or parenteral nutrition, presence of a stoma or gastrostomy
Neurosensory outcomes	1. Visual impairment a. Visual acuity, e.g., total blindness (i.e., no light perception), severe visual impairment, use of glasses b. Cerebral visual impairment 2. Hearing impairment, including response to hearing aids or cochlear implant a. Profound > 90 dB b. Severe 70–90 dB c. Moderate 40–70 dB
Neuromotor outcomes	1. Clinical neurological examination a. Hammersmith Infant Neurological Examination (HINE)^74,79^: 2 to 24 months b. Amiel-Tison Neurological Development from Birth to Six Years^80^: Birth to 6 years 2. Detailed assessment of motor performance a. Gross Motor Function Measure (GMFM)^81^: 6 months to 18 years b. Peabody Developmental Motor Scale, Second Edition (PDMS-2)^82^: Birth to 60 months 3. Diagnosis of cerebral palsy (CP) based on clinical examination and motor function scores, and classified according to the Surveillance of Cerebral Palsy in Europe (SCPE) criteria^83,84^ 4. In children with CP, the Gross Motor Function Classification System (GMFCS) grades severity of motor impairment into 5 levels.^85,86^
Neurocognitive and language outcomes	1. Difficulties in assessing cognition in infants and young children a. Requires attention, some motor function (especially fine motor) to perform tasks, and receptive language to understand. b. There are no established tools for assessment of children who have major sensory or motor impairments. c. At 2 years, there are no established assessment tools for more sophisticated cognitive functions (e.g., executive functions, abstract reasoning) which are still developing. 2. Standardized measures of cognitive and language abilities have been used, but care is required in their interpretation. Translations into many languages not available. Where possible, performance may better be compared to that of typically developing children, although this may not be practicable in the context of a trial. Where direct assessment is not possible, all available evidence should be collected to categorize performance in standard deviation score bands. a. Bayley Scales of Infant Development, Third Edition (BSID-III)^87^: 1 month to 42 months; 5 scales (cognitive, language, motor, social-emotional, and adaptive) b. Griffiths Mental Development Scales, Third Edition (GMDS III)^88^: Birth to 72 months; 5 scales (foundations of learning, language and communication, eye and hand coordination, personal and social-emotional, gross motor) c. Mullen Scales of Early Learning^89^: Birth to 68 months; 5 scales (gross motor, fine motor, visual reception, receptive language, expressive language)
Combined adverse categorical neurodevelopmental outcomes	1. By severity, e.g., British Association of Perinatal Medicine^90^ a. Severe b. Moderate c. Mild 2. For a dichotomous variable, generally children with moderate and severe impairment are combined into a single group (children with neurodevelopmental impairment), with a 2nd group of children with no or mild impairment.
Functional outcomes	1. Mobility, e.g., Gross Motor Classification System 2. Communication: Difficult to assess with standardized assessment tools when there are multiple primary languages 3. Adaptive function: Ability to perform self-help skills (e.g., dressing/undressing, self-feeding) 4. Standardized measures of functional outcome a. Pediatric Evaluation of Disability Inventory (PEDI)^91^: 6 months to 7.5 years; measures self-care, mobility, and social function; targeted for children with disabilities b. Vineland Adaptive Behavioral Scales, Second Edition (VABS-II)^92^: Birth to 90 years; measures adaptive behavior

### School-age children

More precise evaluation of cognitive and neuromotor function beyond the major disabilities is best achieved at school age with the use of standardized age-appropriate instruments. Although for practical reasons drug approval and licensing may be determined based on two-year (corrected age) outcomes, commitment for post marketing surveillance for at least five years should be considered. Processes can be put in place to maintain contact with and trace families to minimize dropout, which could bias trial results (Table 3).

**Table 3:** Secondary outcome measures of school-age children

Criteria	Description
Post-neonatal onset epilepsy	Specify seizure type according to ILAE^77,78^
Neuromotor outcomes	1. Gross Motor Function Classification System (GMFCS) 2. Gross Motor Function Measure (GMFM) 3. Movement Assessment Battery for Children (Movement ABC 2)^93^: 4 to 12 years; targeted to detect gross motor or fine motor impairments 4. Peabody Developmental Motor Scale, revised (PDMS-2)^94^
Cognitive outcomes	1. British Ability Scale, Third Edition (BAS 3)^95^: 3 years to 17 years 11 months; Early Years Battery for 3 years to 5 years 11 months 2. Differential Ability Scales Second Edition (DAS-II)^96^: 3 years 6 months to 17 years 3. Mullen Scale of Early Learning: Birth to 5 years 8 months 4. Stanford-Binet Intelligence Scales, Fifth Edition (SB5)^97^: 2 years to 85 years a. Wechsler Preschool and Primary Scale of Intelligence—Fourth Edition (WPPSI—IV)^98^: 2 years 6 months to 7 years 7 months b. Wechsler Intelligence Scale for Children—Fifth Edition (WISC—V)^99^: 6 years to 16 years 11 months 5. Standardized tests of executive function, which underpin many adverse cognitive outcomes a. NEPSY, Second Edition (NEPSY-II)^100^ b. Behavior Rating Index of Executive Function, Second Edition (BRIEF2)^101^ c. Cognitive Assessment System, Second Edition^102^ 6. Behavior and social-emotional problems: many questionnaires/surveys are available for this age group, including the Child Behavior Checklist^103^, BITSEA^104^, ITSEA^104^, and M-CHAT^105^ 7. Academic attainment tests are for school-age children, 6 years and above

## Drug related issues

Legislation in the EU and USA requires compulsory development of age-appropriate formulations for neonates and children in the development of new drugs. The aim is to support the development of pediatric formulations, which enable neonates to have access to safe, age-appropriate dosage forms. These dosage forms should allow accurate and flexible dose administration and should contain only excipients which are known to be safe and effective for the age of the child.

Further guidance on administration, dosing, and formulation, bioanalytical requirements for drug and biomarker analysis for population PK/PD studies, drug specific safety measures, permitted or prohibited drug specific concomitant care and interventions for ASDs are similar to those for other neonatal drugs^56^ and thus not included in this paper, but are outlined in Supplemental Appendix 2.

## Ethical considerations of ASD trials in neonates

### Ethical challenges in drug development for neonatal seizures

Neonates are uniquely vulnerable clinical research subjects. They deserve special protection, but also deserve to be treated with evidence-based therapies.^12^ The goal of protecting neonates from the risks of research needs to be balanced with the goal of protecting them from non-validated therapies.^11,12^ It is generally agreed that children, including neonates, should be given medicines that have been properly evaluated for their use in the intended population.^57^

Drug trials are scientifically and ethically necessary to establish the efficacy and safety of drugs that are already widely used in neonates as well as to develop new neonate-specific drugs that have not been studied in other populations.^58^ Many challenges of developing ASDs are similar to those for other types of drugs in neonates. However, specific challenges of neonatal seizure trials need to be considered:

A. The timing and nature of acute seizures requiring immediate treatment often allow little time for obtaining informed consent from parent(s).
B. The underlying etiology of the seizures is usually unknown at the time of enrollment or randomization.
C. Seizures are an unexpected medical emergency and are often subclinical, hence the clinical urgency may be difficult for parents to understand.
D. There is a standard of care, namely phenobarbital as first-line treatment,^4^ even though there is little evidence to support its use.
E. Although animal data suggest that treatment of neonatal seizure improves outcome, there is less evidence in human newborns.^1^
F. Epilepsy carries societal stigma which may cause additional parental anxiety.

### Ethical considerations of trial design

The use of placebos in trials is controversial. Since there is a standard treatment for neonatal seizures, it is usually unethical to withhold that treatment, even if the evidence for the efficacy of the standard treatment is weak (e.g., phenobarbital).^24,59^ Guidelines from regulatory authorities in the US and the EU on the use of placebos are clear.^31,60^ Given the expected sequelae of untreated seizures, it would not be permissible to withhold standard therapy, currently phenobarbital. It would be permissible to compare any new ASD to phenobarbital. Placebos could be used to evaluate a new ASD using an “add-on” method, keeping the subjects on identical maintenance treatments with the standard drug (e.g., phenobarbital), then adding treatment with a new ASD to one arm and placebo to the other. This design could also be used to test the initiation of treatment with a new ASD (added on to phenobarbital) at different time points, by having one arm in which the new ASD was started at a defined Time Zero and another arm in which the new ASD was started at some designated later time. The rationale for the use of placebo must be explained in lay language in the consent form and subjects should be told that placebos are only used because of uncertainty regarding whether the new agent is safer or more effective than placebo.

### Ethical considerations of methods of obtaining consent

The full written consent process has been successfully used in many neonatal randomized controlled trials, even those requiring consent within a short time window ( < 6 h, e.g., > 90% consent rate in the original surfactant trial^61,62^ and therapeutic hypothermia for hypoxic-ischemic encephalopathy trial^63^). Full written consent with parental signature should be obtained prior to beginning any research trial of ASDs.^64^ However, neonatal seizures are often both acute and unexpected and may necessitate a much shorter time window during which the treatment should be administered. Informed consent cannot be waived, but there is a provision for exception from informed consent for emergency research, which may be relevant for trials in specific conditions such as status epilepticus in neonates.^65–67^

If parents are not available at the study site for in person consent, the consent could be sent by fax or secure email or with the study hospital’s transport team, reviewed with the study team by phone, and returned by fax, secure email, or by an electronic consent form on a secure website. In addition to obtaining written consent at enrollment, implementation of a continuous consenting process should be strongly considered.^68^ After enrollment, study team members meet with the parents at regular intervals throughout the intervention to ensure that they understand the trial procedures and affirm continued participation. This continuous consenting process is important to supplement the often time pressured review of written consent in neonatal seizure treatment trials.

### Parent participation in ASD trials

Patient and public involvement is important, in particular including parent representatives from parent associations in trial design and implementation.^69^

## Supplementary information

Supplementary information
