# Supplementary material for: Recommendations for the design of therapeutic trials for neonatal seizures
Source: Pediatr Res. 2018 Dec 24;85(7):943–54. doi: 10.1038/s41390-018-0242-2 (PMC6760680; doi:10.1038/s41390-018-0242-2)
Supplement: Supplementary file 1 — Supplementary information [file 41390_2018_242_MOESM1_ESM.docx]

# **Appendix 1**: **Literature Review and International Survey on Neonatal Seizure Treatment to Define Primary Outcome Measures for Neonatal Seizure Treatment Trials**

## A1.1 Methods

### A1.1.1 Literature Review

We reviewed all papers that performed a study of neonatal seizure treatment efficacy and which used EEG or aEEG to assess primary outcome, since neonatal seizures are defined by EEG. An electrographic seizure is defined as a rhythmic stereotyped EEG discharge with a clear onset and offset, lasting for ≥10 seconds, and present in one or more EEG channels that evolves in frequency, amplitude, and morphology.^1^ There is no universal consensus about the definition of status epilepticus in neonates. However, the ACNS terminology defines neonatal status epilepticus when the summed duration of seizures comprises ≥ 50% of any one-hour epoch.^2^ We also reviewed the clinical trials registry, clinicaltrials.gov to determine the primary outcome measure currently in use by ongoing neonatal seizure treatment studies

### A1.1.2 International Survey

We performed an international study of neonatal seizure treatment practice using a questionnaire distributed to specialist neonatal groups and networks of members of the INC. We included questions about specialty, EEG use, years of experience, measures of treatment efficacy, and included some typical case studies of acute neonatal seizures.

### A1.2 Results

### A1.2.1 Literature Review

A recent study performed a number of neonatal seizure treatment trial simulations.^3^ This has established that:

1. A control group is necessary in a neonatal seizure trial as a non-negligible percentage (5–85%) of patients treated with a placebo can fulfill criteria of treatment success due to the natural reduction in neonatal seizures over time.^4^
2. A study protocol needs to proceed very rapidly from patient identification, recruitment and drug administration as soon as practically possible and within a maximum of 24 hours from seizure onset in order to measure drug response or efficacy. If the time from seizure recognition to treatment is too long, then the sample size will need to be increased to measure true efficacy and show a significant difference between groups.
3. In general, the post-intervention seizure burden was the primary outcome measure that resulted in randomized controlled trials with the lowest required sample size, depending on the post-intervention analysis period.

All studies included in the literature review are listed in the table below, and the primary outcome measure used is listed. It is clear from the literature review that while there has been an increase in the number of studies using multichannel EEG to measure treatment efficacy, the following was evident:

- The baseline pre-treatment period was rarely defined.
- The response to treatment was often described as either ‘no response’, ‘complete response’ or ‘partial response’. This partial response was often not defined.
- The most common definition of an acceptable response was ≥80% reduction in pre-treatment seizure burden.
- The time period over which a reduction in seizures should occur following treatment was rarely described but when it was used, a period between 2-6 hours was selected.
- Overall there was no standardized approach to measuring treatment efficacy.

# Table A1: Literature Review of Neonatal Seizure treatment studies using EEG or aEEG monitoring to assess efficacy

**Efficacy/primary outcome data:**

| **EEG or aEEG, and data source** | **Primary endpoint** | **n** | **Clinical and EEG/aEEG or EEG/aEEG alone** | **Drugs used** |
| --- | --- | --- | --- | --- |
| Peer reviewed Literature |  |  |  |  |
| EEG^5^ | Complete control of seizures as shown on EEG or 80% reduction in severity between periods 1 and 3 or 5. | 157 screened  59 had EEG-confirmed seizures | EEG alone for seizure diagnosis and tx efficacy measures.  Clinical seizures  3- electrical, no clinical seizures  48-electrical and clinical seizures | Phenobarbital – 30, phenytoin in 15 phenobarbital-refractory  Phenytoin – 29, phenobarbital in 13 phenytoin-refractory |
| EEG^6^ | Complete absence of seizure activity on EEG or 80% reduction of pretreatment burden | Total – 87  No seizures – 52  Clinical/electrographic seizures – 35 | Clinical only (were given Phenobarbital before starting EEG for trial) – 8  EEG - 27, 5 had to be excluded for protocol violations | 1^st^ line: Phenobarbital up to 40mg/kg (11/22 responded)  2^nd^ line: Clonazepam – 3 (0/5 reached primary endpoint)  Lignocaine – 5 (3/5 reached primary endpoint)  Midazolam – 3 (0/5 reached primary endpoint) |
| aEEG^7^ | “abolished” | 15  11/15-Midazolam abolished seizures | aEEG | Midazolam as third line drug after phenobarbital and lidocaine |
| EEG^8^ | Favorable response to treatment defined as no more than two  electrical seizures per hour of recording lasting less than 30 seconds. | 45 with seizures | EEG alone | Phenobarbital/phenytoin – 32  Midazolam (phenobarb/phenytoin-refractory) - 13 |
| EEG &  aEEG^9^ | Efficacy:  **“good”** – seizures stopped (aEEG and clinical)  **“intermediate”** – diminished seizures/good initial response with a later recurrence (aEEG and/or clinical)  **“no effect”** – no effect (aEEG and/or clinical) | 21 | Drug administered under EEG and aEEG monitoring.  aEEG used to determine Lidocaine efficacy – | 1^st^ line- phenobarbital  2^nd^ line- midazolam (full-term), clonazepam (preterm)  **3^rd^ line- lignocaine;** good = 11/20; intermediate 5/20; none 5/20 |
| aEEG^10^ | Cessation of all seizure activity for 6 hours or longer (both clinical and amplitude-integrated  EEG) was considered a “**good** response”. If seizure activity diminished (sawtooth amplitude-integrated EEG pattern  turned into repetitive single short seizures) or stopped but recurred within less than 6 hours, it was considered as a  **“partial** response”. | 30  2^nd^ line Lidocaine 22; 11-good response, 6-partial response  3^rd^ line 5-good response  2^nd^ line Midazolam  8; 4-partial response  3^rd^ line 4; 2-good response, 2-partial response | Treatment efficacy- aEEG | 1^st^ line- phenobarbital and IV bolus of benzodiazepine  **2^nd^ line – lidocaine and/or midazolam**  **3^rd^ line – whatever was not used second line** |
| EEG^11^ | Seizure termination OR >50% reduced are the two measures reported | 23  8: seizure control in 24 hours (7 abolished, 1 >50% reduced) 4: seizure control in 24-72 hours (2 abolished, 2>50% reduced) | EEG | Levetiracetam  First-line in 4, second-line in 14 (PB first) and third-line or later in 5 (PB and PHT first) |
| EEG^12^ | Seizure cessation (time to seizure cessation) | 22  19/22 had immediate seizure cessation at 1 hour post dose.  Complete cessation:  7/22 by 1 hour  14/22 by 24 hours  19/22 by 48 hours  22/22 by 72 hours | Clinical and EEG | Levetiracetam  All but 3 received it second-line or later |
| EEG^13^ | Seizure cessation  No additional ASDs needed | 18  6/18 had seizure cessation and no need for additional ASDs  Additional 3/18 had initial response, but seizures recurred | Clinical and EEG | Levetiracetam |
| aEEG or EEG^14^ | Efficacy of lidocaine at terminating neonatal seizures  **Complete:** clinical cessation of seizures during treatment and cessation of seizures on aEEG/EEG or improvement in epileptiform activity on sequential EEG s and absence of subsequent additional ASD treatment for the purpose of seizure control.  **Probable**: clinical cessation of seizures during treatment  in the absence of aEEG/EEG monitoring (or inconclusive /invalid for assessment) and absence of subsequent additional ASD treatment for the purpose of seizure control.  **Incomplete:** not fulfilling criteria of above categories. | 30  16-complete  3-probable  11-incomplete | aEEG or EEG as per primary outcome | Lidocaine as second-line treatment without prior phenobarbital treatment |
| EEG^15^ | Seizure cessation in preterm (time to seizure cessation) | 11  9/11 seizure cessation by 24 hours  Complete seizure cessation  4/11 by 1 hour  9/11 by 24 hours  10/11 by 48 hours and 72 hours | Clinical and EEG | Levetiracetam  All but three received it second-line |
| aEEG^16^ | Responsiveness was defined as a reduction of electrographic seizure burden of >80%, and no third-line anticonvulsant drug being required during infusion. | 22  5-seizure control with midazolam  17-needed rescue antiepileptic | aEEG | Midazolam for phenobarbital-refractory seizures |
| EEG^17^ | Reduction in electrographic seizure burden of more than 80% without the need for rescue antiepileptic drugs in more than 50% patients (NEMO) | 14  5/14 met seizure reduction endpoint  Only 2/14 met seizure reduction endpoint and did not need additional ASDs | EEG | Bumetanide as adjunct to PB |
| aEEG^18^ | **Good response:** cessation of seizure activity on aEEG and no recurrence of seizure activity on aEEG for >4 h, after which only single short seizures were observed in some infants, but without the need for rescue ASD, in keeping with a previous study on lidocaine responsiveness, after consensus agreement in the NEMO consortium.  **Intermediate response**: cessation of seizure activity on aEEG, but recurrence of repetitive seizures within 2–4 h for which rescue ASD was given; cessation of seizure activity on aEEG for >4 h, but recurrence of repetitive seizures within 24 h for which rescue ASD was given.  **No response**: seizure activity continued unchanged on aEEG. | 413  319-full-term  94-preterm | Treatment efficacy- aEEG alone | Lidocaine as 2^nd^ line or 3^rd^ line |
| EEG^19^ | Primary: Seizure burden  Secondary: Time to seizure cessation |  | Two arms:   - Clinical only - EEG only | Prompt tx with PB, PHT and MIDAZ |
| EEG^20^ | Complete (cessation of clinical and electrographic seizures), partial (reduction of electrographic seizures with first bolus & response to second bolus),  or no response (no response after second bolus)  NSE excluded | n = 91  62.6% (57) – complete response  16.5% (15) – partial response  20.9% (19) – no response | EEG | Phenobarbital |
| **Studies ongoing from Clinical Trials.gov** |  |  |  |  |
|  |  |  |  |  |
| EEG  NCT01720667 Efficacy of Intravenous Levetiracetam in Neonatal Seizures (NEOLEV2) | Seizures terminated (measured in terms of seizure burden) |  |  | IV Levetiracetam vs IV Phenobarbital |
| EEG  NCT02229123 Levetiracetam Treatment of Neonatal Seizures: Safety and Efficacy Phase II Study (LEVNEONAT1) | Seizure burden reduced by 80% during 3^rd^ and 4^th^ hour after LVT admin. compared to 2 hours before LVT AND  No need for rescue ASD within 48 hours |  |  | Levetiracetam |

Sedighi et al., Neurosciences (Riyadh), 2016. *Efficacy and safety of levetiracetam in the management of seizures in neonates***. Not included in table as EEG only carried out after 7, 30 and 90 days.**

## A1.2.2 International survey results

There were 87 respondents with a median of 15 years (IQR 8-28) experience treating neonatal seizures; 51% were neonatologists, 39% were pediatric neurologists and the remainder from other specialty groups. Most respondents (87%) reported treating seizures with an EEG correlate irrespective of an accompanying clinical correlate, and most (80%) considered the seizure burden to be the most important factor when deciding to treat (i.e. combined seizure number and duration). There was considerable variability in the seizure burden threshold used to commence treatment. Neonatologists were more likely than non-neonatologists to require longer seizures or clinical correlates to start treatment but 65% overall would treat ≥2 seizures/hour, >50% would treat a seizure lasting >1 minute, and 38% would treat single seizures of at least 10 seconds duration. With regard to treatment success, 70% responded that complete abolition of seizures in the EEG was the ideal success measure and over 80% expected a response within 1 hour of drug administration. In response to specific case studies, most respondents would be willing to randomize a neonate to a study drug if short electrographic seizures re-emerge following a standard loading dose of phenobarbital. Full survey results are available on the INC website here: <https://c-path.org/programs/inc/>

# **Appendix 2**: **Drug-Related Issues**

1

# Appendix 2A: Dosing and Administration guidance

**Table A2-1 - Preferred routes of administration and formulation options as a function of the child’s age highlighting applicability / preference (1 (red) = low, 5 (green) = high)***

| **Route**  Dosage Form | **Preterm newborn infants** | **Term newborn infants (0d-28d)** | **Infants and Toddlers (1m-2y)** |
| --- | --- | --- | --- |
| **Peroral** |  |  |  |
| Solution/ Drops | 2 | 4 | 5 |
| Emulsion/ Suspension | 2 | 3 | 4 |
| Effervescent DF | 2 | 4 | 5 |
| Powders/ Multiparticulates | 1 | 2 | 2 |
| Granules/Pellets | 1 | 3 | 4 |
| Tablets | 1 | 1 | 1 |
| Melting Tablets | 1 | 1 | 3 |
| Mini-Tablets (<3mm) | 1 | 1 | 3 |
| Capsules | 1 | 1 | 1 |
| Orodispersable DF | 1 | 2 | 3 |
| Chewable tablets | 1 | 1 | 1 |
| Melt-away films/wafers | 1 | 2 | 3 |
| Sustained-release films/wafers | 1 | 1 | 2 |
| *Fast-dissolving drug formulations* |  |  |  |
| Orally disintegrating tablets | 1 | 3 | 4 |
| Lyophilisates | 1 | 3 | 4 |
| Flash-release films/wafers | 1 | 3 | 4 |
|  |  |  |  |
| **Nasal** |  |  |  |
| Solution | 3 | 4 | 4 |
| Semisolid DF | 2 | 3 | 3 |
| **Rectal** |  |  |  |
| Suppositories | 4 | 5 | 5 |
| Rectal Enema | 5 | 4 | 4 |
| Rectal capsules | 2 | 3 | 4 |
| **Topical/ transdermal** |  |  |  |
| Ointment, Cream, Gel | 4 | 4 | 4 |
| Liquid DF | 4 | 4 | 4 |
| Transdermal Patch | 1 | 2 | 2 |
| **Parenteral** |  |  |  |
| i.v. Solution | 5 | 4 | 4 |
| i.m. | 3 | 3 | 3 |
| s.c. | 4 | 4 | 4 |
| Pump system | 5 | 4 | 4 |
| **Ocular** |  |  |  |
| Eye drops | 3 | 4 | 4 |
| Semisolid DF | 2 | 3 | 4 |

* adapted from J. Krause, J. Breitkreutz, Pharm. Med. 22: 41-50 (2008)

**Peroral administration and dosage forms**

The oral route of administration is commonly used for dosing medicinal drug products to children and consequently many drugs are developed as solid or liquid dosage forms. For all oral dosage forms, acceptable palatability is critical for compliance and adherence.

**Powder or Granules for reconstitution**

Ready-to-use solutions & suspensions are challenging to develop. Frequently sufficient shelf life can only be produced when large quantities of excipients are present and in most cases in quantities that are harmful to children. Powder or granules for reconstitution as a suspension or solution offer a suitable alternative formulation approach, which can often utilize the technical stability information generated from the adult dosage form and still provide a liquid formulation at point of administration. Depending on the dose and the corresponding volume to be administered, it may be necessary to switch to an alternative solid dosage form (DF).

Whether a reconstituted solution or suspension is created depends on the DS solubility and the excipients employed. In addition to the in-use requirements of a solution and suspension formulation (chemical, physical and microbial stability) bulk stability of the powder/granules needs to be assured to enable a suitable drug product shelf life. Powders/granules for reconstitution may require the use of specific excipients to produce free-flowing powder/granules with sufficient content uniformity. The primary packaging also needs to be considered when developing powder/granules for reconstitution and as such the filling principle needs to be taken into consideration during technical feasibility trials. Powders/granules can be dispensed in capsules, sachets / stickpacks, or bottles (typically known as Powder in Bottle) and this will typically depend on the dose and/or the amount of powder/granules required to achieve a reconstituted formulation and/or whether the product is intended for single use (e.g. dispersible) or multi use (e.g. 2 week reconstitution). Preference should be given to ready to use formulations.

Palatability plays an important role when developing these dosage forms for most children although likely not as great a consideration for the neonate specifically. Taste is a particular challenge as there will be a limited quantity of sweeteners and flavouring agents that may be incorporated into this dosage form in order to maintain a certain fill volume. The use of insoluble salt forms of the drug and/or taste masking technologies, such as particle coating, may be considered to improve palatability. It should be noted that while these strategies reduce dissolution in the mouth and hence taste perception, they might affect the pharmacokinetic profile of the drug.

Less applicable for the neonate includes addition of powders/granules that can be reconstituted effervescent dosage forms, dispersible dosage forms and oro-dispersible dosage forms that can be reconstituted. Liquid formulations include solutions, syrups, suspensions (ready-to-use or for reconstitution), emulsions, and micro-emulsions. Elixirs contain a high percent of ethanol and, therefore, are not recommended for pediatric use. Typical target dose volumes for pediatric liquid formulations are < 5 mL for children under 5 years and < 10 mL for children of 5 years and older. However, the more palatable the formulation, the higher the dose volume which will be tolerated. To limit the dose volume given to neonates and infants, concentrated drops may be useful providing the formulation is palatable.

**Extemporaneous liquid dosage forms**

A crushed tablet, pharmaceutical intermediate (e.g. granulate) or the contents of a capsule can be added to a palatable vehicle immediately prior to use to produce an extemporaneous liquid formulation (ELF). ELFs enable rapid, pragmatic development of a compound that already has a solid dosage form developed, therefore, reducing development timelines. ELFs allow flexibility in dose selection because doses can be adjusted based on real-time cohort data and allow precise dose control that is important for early clinical evaluation where the therapeutic index is still being established. This concept also enables flexibility in the patient cohort where the drug is administered, e.g. pediatric patients where non-standard doses are more easily and accurately measured by using a liquid formulation, adults unable to swallow tablets or capsules and patients receiving medicines via nasogastric or gastrostomy tubes.

**Minitablets/other multiparticulate systems (Not yet established in the neonate)**

For drugs that are not stable in aqueous media or cannot be taste masked in liquid preparations, minitablets (multiple unit dosage < 3.0 mm in diameter) and other multiparticulate formulations (e.g. beads, granules, powders) should be considered. These dosage forms might also be able to utilize synergies from the adult tablet/capsule development if applicable e.g. similar final blend composition and manufacturing process. Multiparticulate dosage forms can be dosed directly into the mouth of the infant or child, or by mixing the prescribed dose with a small amount of soft food or with breast milk/formula prior to administration. A modification of the dosage form principle would deem the multiparticulates as sprinkles. Formulations can be provided in a bottle with dosing scoop, in single-dose sachets/stickpacks or contained within capsules at various fixed doses, which can be opened prior to administration.

The product information should specify which commonly available foods/drinks are suitable for mixing with the preparation, list foods/drinks that should be avoided due to stability, compatibility, bioavailability or taste issues and as such compatibility with food/beverage is an important aspect of formulation development for minitablets / multiparticulate systems.

Selection of minitablets instead of reconstitution preparations can be considered when:

- The API has a very aversive taste or is instable in water
- There is inadequate physical/chemical/microbial stability of the reconstituted formulation
- A liquid formulation is suitable for the target ADME profile of the API
- A modified release profile is required
- The target profile is a fixed dose combination
- The dosing volume is too high for liquid administration
- Storage in a refrigerator has to be avoided.
- A solid dosage form is required (WHO, UNICEF request)
- The filling of the powder / granules into the primary packaging is not technically feasible or compromises the quality (e.g. content uniformity)

Minitablets are not a viable option if:

- Number of tablets is too high (acceptability depends on number mini tablets given as one dose)
- Dosing increments can’t be met
- Administration via nasogastric tube is required and the mini tablets can’t be dispersed into a liquid
- Individual dosing (1 unit) is required and the drug load is low (content uniformity issue)

As this protocol is specifically targeted for the neonate, issues that are critical for infants and older children play less of a role. These factors include taste or taste masking of oral solutions, coloring, texture, fillers and coatings and thus will not be addressed here in detail. There are however new techniques using taste aversion in animal models, adult taste panels and use of the electronic tongue (e-tongue) that can give basic assessment of acceptability before use in the neonate.

**Topical Delivery**

The topical route of administration is used to treat local skin diseases, usually avoiding systemic effects. Special care should be considered when developing a topical formulation for the pediatric population. Indeed, important morphological and thus permeability differences exist between normal (mature) skin and that of a neonate (especially the preterm infant). The stratum corneum is thin in neonates and will reach normal thickness 10-30 days after birth. The epidermis is perfused and hydrated to a greater extent than in adults. Infants and young children have a larger body surface area to weight ratio than adults. Substances can be variably absorbed through the skin and reach higher blood and tissue levels and may result in toxicity (e.g. adrenal suppression with topical corticosteroids).

**Parenteral administration**

For seriously ill neonates, intravenous (IV) drug administration is preferable. If possible, drugs are injected through an indwelling venous line. Peripheral veins with comparatively slow blood flow will be irritated by a high osmotic load, extremes of pH, and the chemical nature of some active substances and excipients. Sclerotisation, thrombo-phlebitis or infiltration of the tissues may result with loss of the vein for therapy and possibly tissue damage.

Blood flow in central veins is fast; therefore drugs administered via a central catheter are more rapidly diluted and distributed. Intravenous formulations administered by the central route do not always need to be diluted to the same extent as those given into peripheral veins. Slow injection of an undiluted product into a running infusion may also be considered provided that compatibility has been evaluated. The ability to give more concentrated drugs may be very important when fluid intake is restricted. However, the rate of administration should take into account the potential for toxicity and adverse effects.

**Formulation considerations for intravenous administration**

**Reconstitution volumes**

If the drug product is a lyophilized powder, the total volume after reconstitution should be an even number, which simplifies calculation of proportions in the likely case that only a part of the dose for adults is to be administered. Reconstitution instructions should state the exact volume to be added and the resulting dose per volume after reconstitution to enable the healthcare giver to do a proper dose calculation.

**Osmolality and pH**

Careful consideration must be given to the concentration and nature of excipients. Excipients may contribute to the body’s osmotic load and cause serious systemic adverse effects in sick neonates. Hyper-osmolar injections and extremes of pH may irritate small peripheral veins and cause thrombophlebitis and extravasation. Hypo-osmolar injections may induce hemolysis. An iso-osmotic solution, including agents that do not decrease drug substance solubility, is preferred and should be achieved by adding suitable tonicity adjusting agents such as sodium chloride or glucose to the formulation or by recommending appropriate dilution prior to administration. Hyper-osmolar injections may be appropriate for central venous administration without further dilution. In this case the rate of administration should be limited.

Infusions should have a low titration acidity or alkalinity and be preferably of neutral pH and iso-osmotic at concentration of administration. Clear warnings must be given about the need for dilution or co-infusion and regarding compatible infusion solutions.

**Volume**

Careful consideration must be given to the volume of injections and infusions. The acceptable daily intake of fluid for neonates receiving intravenous therapy is related to age and weight. For example, a 1 kg neonate may only receive 150 ml per kg day which includes all nutritional requirements as well as therapy. The volume and electrolyte content of intravenous infusions and injections may contribute critically to the daily intake. The sodium requirement of neonates is approximately 3 mmol/kg/day (depending on gestational and chronological age). The need for dilution and catheter flushing must also be carefully assessed in relation to osmolality, pH and chemical irritancy of the active substance, as well as the excipients used. When making recommendations for dilution and flushing of intravenous drugs one should take into consideration that several intravenous drugs may be required when pediatric patients are seriously ill. The smallest practical volumes should therefore be quoted. Residual volumes in giving sets and intravenous lines may be significant for neonates, and special low volumes medical devices for this purpose should be considered.

**Excipients**

The choice of excipients used in pediatric formulations has to be carefully considered and the function and quantity needs to be justified to the Health Authority. Some excipients maybe restricted or completely banned in individual countries / globally when the intended use is for a pediatric population. Although excipients should be pharmacologically inactive they may indeed cause adverse effects themselves, particularly in pediatric patients, as such the tolerability of excipients varies across the pediatric age range. It has to be noted that the physiology of neonates differs considerably from that of older children and adults. They may not be able to metabolize or eliminate an excipient in the same manner as an adult and as such the quantities of excipients need to be age-appropriate or in some instances the excipient avoided altogether. At present there is very little information on the acceptable daily intake (ADI) of all the excipients in pediatric patients. The only useful guide for formulation development is to use the adult ADI, which is based on a 70kg adult and extrapolate down to the weight of the target population (certainly not optimal). There is also an external resource known as the Safety and Toxicity of Excipients for Pediatrics (STEP) database, which is of great benefit and should be consulted where applicable (<http://www.eupfi.org/step-database/> ). Other organizations working on pediatric formulations may also be consulted including the regulatory web sites for EMA and FDA, EUPFI (European Pediatric Formulation Initiative [www.eupfi.org](http://www.eupfi.org)) and Pediatric Formulation Initiative of NICHD BPCA program.

The preferred dosing vehicle should include the following excipients: suspending agent (e.g. CMC), antioxidants to increase chemical stability of DS, flavours / sweeteners to improve palatability, as well as preservatives to minimize microbial growth during storage and a buffer to maintain pH. The need for each of these components must be carefully considered for the neonate.

**Preservatives**

All multi-dose containers must contain preservatives to guarantee microbial stability of the DP during shelf-life. The preservative selected should inhibit the microorganism growth in the DP. Typically, most preservatives exert their effect in the pH range 4-7. Examples include methyl paraben, propyl paraben, sorbic acid, and sodium benzoate. Recent publications indicated that propyl paraben is not suitable for neonates and infants and its usage is to be avoided where feasible. The final choice will be based on excipient compatibility, preservative solubility in DP, and anti-microbial effectiveness testing (AET).

A special effort should be made on using the lowest preservative concentration as possible, to avoid the probability of allergic reactions to preservatives. The DP pH range should be evaluated with the chosen preservative system for AET testing. The preservative system should be challenged with 90%, 75% and 50% of target concentration with AET testing. The justification for the minimum amount of preservative needs to be evaluated considering also the product at the end of shelf life.

WARNING: It is mandatory to avoid any association of benzoates with vitamin C (anti-oxidant) which leads to benzene formation (carcinogenic).

**Co-solvents**

Commonly used co-solvents such as ethanol and propylene glycol require special attention when formulating for neonates. Depending on the age of the child, these excipients may have contra-indications. Severe acute and chronic concerns exist with ethanol. Concerning propylene glycol, children below 4 years have a limited metabolic pathway (alcohol dehydrogenase), which can lead to accumulation of propylene glycol in the body. High concentrations of sorbitol may have an osmotic effect, leading to excessive diarrhea.

**Table A2-2 - Summary table of excipients with potential toxic risk***


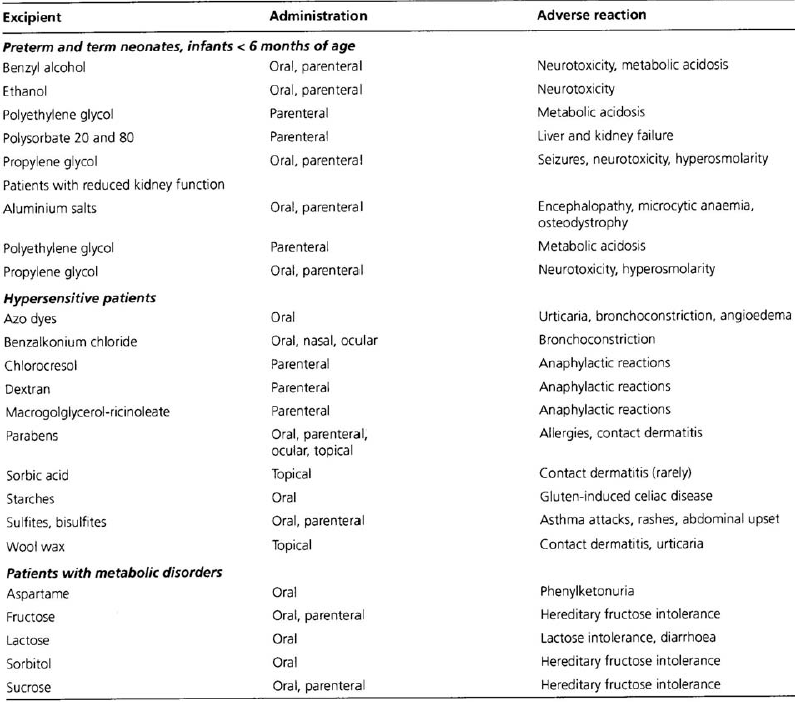


**Placebo use:** while placebo use may be less common in the neonate, if it is used, the formulation needs to be indistinguishable from the test compound particularly for blinding the investigators, nurses and parents.

Appendix 2B Age appropriate formulations including excipients

### A2B.1 Introduction

Legislation passed in the EU and USA requires compulsory development of age-appropriate formulations for neonates and children. The aim is to support the development of pediatric formulations, which enable neonates to have access to safe, age-appropriate dosage forms. These dosage forms should allow accurate and flexible dose administration with excipients that are known to be safe and effective for the age of the child. The following initial information should be available:

- Target population (gestational and postnatal age)
- Drug administration route
- Dose/dosing regimen including dosing flexibility
- Dosing devices
- Drug absorption site (e.g. gastric, intestinal)
- Drug-Food interactions
- Drug-Drug interactions (e.g., CYP 3A4 inhibition)
- Development strategy
- Timelines/ Availability of Toxicology data
- Availability/ Timelines of adult PK/ safety/ efficacy data

### A2B.2 Specific consideration for neonates

There is no single formulation that is ideal for all neonates. As such, a range of appropriate dosage forms for a particular compound may be required. The following are important considerations:

- Minimal dosage frequency
- Minimal volume of usual neonatal dose
- Minimal impact on lifestyle (in-patient vs. home administration)
- Stability in age appropriate formulations
- Non-toxic excipients
- Convenient, easy, reliable for accurate dose administration
- Technical complexity of manufacturing or where no commercial formulations exists – extemporaneous preparation need to be defined
- Packaging and device options
- Pre-clinical development (safety assessment) in juvenile animal studies
- Juvenile animal studies are useful to predict the dose in children, for risk minimization and for the identification of safety parameters to monitor and detect early safety signals. The species and age range tested will depend on the drug class and proposed target population. Only a limited database for toxicology studies in very young animals is currently available and sometimes unique toxicities can be found in the pediatric population which have not been observed in adults.^21^ The Health and Environmental Sciences Institute (HESI) of the International Life Sciences Institute (ILSI) is examining nonclinical models for pediatric drug development^22^.

Neonates will often require additional juvenile studies, as PDCO often lowers the age limit of the target pediatric population to produce scientifically sound data in the most vulnerable populations and subsequently prevent later off-label use in infants and neonates. Neonates and infants have immature organ systems and special attention will be paid to data generated from juvenile animal studies.

During early development of new drugs intended for oral administration, the main emphasis is on suitability of compound properties for adult dosage forms, with the intention of developing tablet or capsule formulations. Although solid dosage forms are widely accepted by older children and adolescents, younger children and neonates will require liquid formulations at point of administration.^23,24^ Notably, initial treatment of neonatal seizures will usually be by the intravenous route.

The taste of a drug is not considered when nominating new compounds for development. Reasons include:

- The most relevant selection criteria are safety, tolerability and efficacy of the compound, which are based on non-clinical testing and physico-chemical properties such as solubility, permeability, stability and crystallinity.
- Adult oral dosage forms can be easily taste masked by encapsulation or film coating techniques.
- There is a lack of robust and reliable techniques for early taste assessment of compounds when only limited toxicity data is available.

In addition, the molecule’s solubility characteristics may not be ideal for the liquid dosage form approaches needed for infants and young children. Compounds with high solubility can be difficult to taste mask in liquid preparations, as they often cannot be formulated as suspensions. Even suspensions of poorly soluble compounds can exhibit poor palatability characteristics if the small amount of solubilized drug exceeds the human taste threshold or if mouthfeel is compromised by the suspended drug substance.

The proposed administration, dosing and formulation including excipients in the study drug needs to be defined. Further guidance on this topic is outlined in **Appendix 2 Dosing and Administration Guidance.**

Dose of administration

The protocol must provide instructions on the following points:

- Provide details of doses to be given including placebo, use mg(g)/kg body weight if appropriate
- Consider loading and maintenance dose, if appropriate
- Specify route and frequency of administration e.g. intravenous, intramuscular, oral, rectal, buccal and sublingual.
- Define any immediate safety observations needed: changes in heart rate, respiratory rate, blood pressure, oxygen saturation.

## A2B.3 Measures of drug levels for adequate analysis of PK/PD

## A2B.3.1 Bioanalytical requirements for drug and biomarker analysis for population PK/PD studies

A crucial requirement for any pharmacokinetic-pharmacodynamic (PK/PD) study is the availability of robust and validated analytical methods (assay). The assay has to be capable of reliably detecting a given analyte (the parent compound and/or metabolites and biomarker) over a wide concentration range to describe the PK/PD time course in a biological matrix with sufficient specificity, sensitivity, accuracy, and precision. For this reason all PK and biomarker laboratory techniques, assays, and procedures to be used as part of a PK/PD study will have to be developed and validated according to the most recent regulatory guidance. ^25–27^

This document provides a brief summary of assay requirements and validation steps while referring to the relevant guidance documents for specific details.

## A2B.3.2 Bioanalytical validation

A bioanalytical procedure describes the requirements for a full method validation. Full validation includes the determination of the accuracy, precision, lower level of quantification (LLOQ), analyte stability, analyte recovery, selectivity, limit of detection (LOD) and calibration range. Full validation is preformed to demonstrate that a particular method used for quantitative measurement of analytes in a given biological matrix is reliable and reproducible for the intended use. Detailed procedures for individual methods are described separately in standard operating procedures for each assay. A full validation is performed when developing and implementing a bioanalytical method for the first time. A partial validation is a modification of an already validated and/or published bioanalytical method that does not call for full validation. Partial validation can range from one intra-assay accuracy and precision determination to a nearly full validation. If intended for regulatory submission, it is important to appropriately document all method validation and sample analysis activities, including using audit trails for instruments.

The specific recommendations for methods development and validation are presented in the referenced guidance documents. In general the elements contained in Table 4 have to be established for each method.^27–29^ In addition the method has to be accurate for microanalytes since the amount of available bioanalytical sample in neonates if limited.

**Table A2-3: Elements to Establish for Each Method**

| Elements | Description | |
| --- | --- | --- |
| Stability | Conditions to ensure stability of analyte or antigen in matrix during collection, processing, storage and analysis |  |
| Linear range | Range over which procedure has been demonstrated to produce a reproducible, linear response (reproducible, nonlinear response may also be acceptable). |  |
| Specificity | Ability of a method to measure only what it is intended to measure. |  |
| Sensitivity | Limit of detection, limit of quantitation, and 95% confidence interval of standard curve. |  |
| Inter-day & intra-day accuracy | Closeness of determined value to true value. |  |
| Inter-day & intra-day precision | Variability of replicate determinations. |  |
| Detection limit & Lower limit of quantification (LLOQ) | Determination of signal to noise ratio. LLOQ = lowest amount of an analyte that can be quantitatively determined, reproducible with precision of 20 % and accuracy of 80-120 %. |  |
| Ruggedness | Variability between operators, instrument, columns, *etc*. |  |
| Additional elements include | Extraction recovery, matrix interference, dilution integrity and ion suppression (for assays with mass spectrometry detection) |  |

## Appendix 2C. Population Pharmacokinetic Modeling

There are two basic approaches for performing pharmacokinetic evaluations: traditional pharmacokinetics and population pharmacokinetics (PK). Traditional PK studies involve administration of either single or multiple doses of a drug to a relatively small group of subjects (e.g., 6-12) with relatively frequent blood sample collection.^30,31^ In the past, pediatric PK data often came from small underpowered studies that applied variations to traditional serial sampling strategies to characterize patients’ individual profiles. Given the challenges of performing studies in neonates and traditional PK studies using pediatric unfriendly designs burdened by intense sampling schedules and long waiting times (at least one dosing interval), enrollment of sufficient subjects was often an issue. As a result, studies would typically recruit fewer subjects than required to generate statistically robust information on factors explaining PK/PD variability between patients in the same age category, as well as across the age spectrum from neonates to adolescents.^32,33^ Thus traditional PK studies are unlikely to be feasible in neonates and should be avoided.

The introduction of the ‘population approach’ in neonatal studies has provided more flexibility while retaining good information content for parameter estimation.^34,35^ A population PK/PD study does not require fixed and intense serial sampling but can accommodate imbalanced designs with sampling across different subjects and time intervals. Sparse sampling with limited number of observations per subject is preferred, or a combinations of both strategies can be used. The non-linear mixed effect modeling technique allows the estimation of the typical values of PK parameters and the within and between subject variability. In addition, the modeling allows the identification of patient characteristics that are associated with variability in PK parameter estimates such as age, gender, body weight and size, race and ethnicity, liver and renal function, and genotype (referred to as covariates).^36,37^ The application of population PK/PD analysis has opened new avenues for pediatric drug studies and has resulted in many successful PK/PD studies conducted by the NICHD Pediatric Pharmacology Research Unit Network using this approach.^38^ The trial investigators (and ideally the DSMB) should include a pharmacologist with expertise in pharmacokinetic study design and analysis in the neonatal population.

Further guidance on different approaches to pharmacokinetic modelling is given below - in **Guidance on Pharmakokinetic Modeling**. The chosen method should be described in the study protocol.

**Guidance on Pharmakokinetic Modeling**

**Nonlinear Mixed Effect Modeling (NONMEM)**

This method of population modeling was first introduced in the late seventies by Sheiner and Beal.^39,40^ The acronym NONMEM which stands for Nonlinear Mixed Effects Modeling has become synonym to ‘population approach’, but it also refers to the computer package that initially was made available by this group. To date there are several software packages that provide nonlinear mixed effects modeling capabilities. ^41,42^

With the population approach all responses from all subjects are analyzed *simultaneously*. In addition, balanced as well as unbalanced data can be simultaneously analyzed with different subjects contributing varying amounts of data. This provides flexibility and allows the analysis of both rich and sparse experimental data. A parametric population analysis estimates the central tendency or ‘typical’ (i.e. population mean or median) of the parameters of a user specified structural model (e.g. clearance and volume), between-subject variability, and residual variability (Table 1). Mixed effects models consist of fixed effect and random effect parameters. Typical fixed effect parameters are clearance and volume and the predictive relationships between these parameters and clinical and pharmacogenetic/genomic factors (i.e. covariates). For example between-subject variability in clearance may be partly predicted by body size (i.e. allometrically scaled weight) and creatinine clearance (Table 1). Parameters are assumed to be normally (or log-normally) distributed where η is a random variability with a mean of 0 and a variance of ω^2^ (omega squared). Epsilon is the term NONMEM uses to quantify residual variability. As a particular ε cannot be defined (as it is random), its distribution is assumed to be normally distributed, with mean 0, and variance σ^2^ (sigma squared).^43^

Although there is not one universal method to develop population models, the general steps involved are as outlined below.^34,44–49^

Step 1: Establishing a database. Data collected in clinical studies are often complex and require extensive cleaning and reformatting. Accurate information is required on patient’s demographics, dosing, timing of dose events, sampling of drug concentration and other biomarkers, additional laboratory measurements and clinical status. Meticulous checking and cleaning of the data before the actual modeling is one of the most important and time consuming first steps and is part of good modeling practice.

Step 2: Base model. The structural PK models can be defined as one-, two-, or three-compartmental models. For orally administered drugs parameterization typically is with a first-order (Ka) or zero-order rate constant for absorption, combined with clearance (CL/F), inter-compartment clearances (Q/F) and apparent volume of distribution(s) (V/F). Typically data are log transformed (natural logarithm) and modeled using the first-order conditional estimation method with interactions. Between-subject variability (BSV) and between-occasion variability (BOV) are modeled exponentially. Residual error is modeled as additive, a constant coefficient of variation or a combination of the two (Table 1).

Step 3: Covariate analysis. Potential covariates are investigated using a stepwise forward addition and backward elimination process. A significance level of 0.05 is typically used in the forward step to allow inclusion of potential covariates. More stringent criteria are used in the backward elimination step (e.g. P-values of 0.01 or 0.001) to retain only clinically meaningful covariates in the final model.

Step 4: Model validation. The difference in objective function value (ΔOFV) computed as -2×loglikelihood is used as the statistical criterion for differentiation between hierarchical models. Further assessment and comparison uses the likelihood ratio test and evaluates changes in the objective function value (OFV) between models. Improvement in model fit is determined using the chi-squared distribution with one degree of freedom (ΔOFV <3.84 = p <0.05). Models are also compared using the Akaike information criterion (AIC) and Schwarz information criterion (SIC) to discriminate between non-hierarchical models in the selection of a structural model. Goodness-of-fit plots and simulation-based diagnostics are used for model evaluation. Numerical- and visual predictive checks are used to assess the predictive performance of the final model. The final model with variability included is then used to simulate outputs (e.g. concentrations) at each sampling time-point and the 95% confidence interval around the simulated 5th, 50th and 95th percentiles are overlaid with the same percentiles of observed data to evaluate the predictive power of the model (visual predictive check). The percentages of observations below and above the simulated 5th and 95th percentile can also be calculated for a numerical predictive check. A non-parametric re-sampling bootstrap analysis to assess model accuracy and stability is performed to verify relative standard errors and confidence intervals of the final population parameter estimates.

Of note, there are other population modeling programs besides NONMEM. The nonparametric expectation maximization algorithm (NPEM) first described by Schumitzky^50^ has undergone several iterations and is now as a nonparametric adaptive grid (NPAG) the basis of the Pmetrics package to perform non-parametric and parametric pharmacokinetic-pharmacodynamic population and individual modeling and simulation.^51,52^Another software platform for the analysis of nonlinear mixed effects models is Monolix (The software Monolix for parameter estimation: [www.lixoft.eu](http://www.lixoft.eu/)).

**Sample size justification**

Sample size justification is important prerequisite for pediatric PK and population PK/PD studies. Yet, there is not a well-developed paradigm for this.^53^ Even to date, many population analyses in the literature or submitted for publication whether in pediatric patients or adults do not mention sample size considerations. Often sample size is driven by the patients and number of observations that could be included rather opportunistically over the study period. A recent publication of precision criteria to derive sample size for pediatric studies has renewed the discussion related to regulatory guidance for standardization but no consensus exists.^54^ The proposed framework is viewed as a reasonable starting point. However, other methods (e.g. simulation based; or including clinical covariates other than age) have been proposed for sample size justification. Clinical trial simulation is also used to estimate appropriate sample size and study power.

**Optimal sampling strategies**

Limiting the number of concentration measurements while maintaining accuracy of PK parameter estimates is of importance for practical, ethical, and economic reasons.^55^ Sampling strategies are formulated to reduce the number of samples but maintain adequate accuracy. A diversity exists in the literature regarding sampling strategy nomenclature. Terms such as limited, optimal, minimal, and sparse sampling. An attractive option is to include optimal sampling strategies in order to maximize the information content for the characterization of a drug PK behavior in infants and children while minimizing the number of blood draws and the total blood volume to be collected.

David D’Argenio was one of the e first to apply a determinant (D)-optimal sam­pling design to pharmacokinetic studies,^56^ and in recent years multiple studies have indicated that resul­ts in indivi­dual pharmaco­ki­netic parame­ter esti­mates were as good as those obtained with traditio­nal data-rich sets.^56,57^ Traditional D-optimal design theory has the property of replication with the explicit assumption that there is one true parameter vector. With D-optimality as the design criterion, the determinant of the inverse Fisher information matrix is the scalar that is being optimized. This results in the number of optimal samples being identical to the number of system parameters. One of the earlier software packages for the estimation of optimal sampling times is the ADAPT II package of programs of D'Argenio and Schumitzky (D. Z. D'Argenio and A. Schumitzky, user's manual for ADAPT II, Biomedical Simulations Resource, University of Southern California).^58^

More recent population D-optimization approaches can be performed using programs such as WinPopT and PFIM.^59,60^ The application of these techniques will help in reducing the number of samples per subject as well as reducing the number of subjects required to ensure a most informative population based modeling design.

**Table A2-4.** Equations for a simple population model as used in NONMEM

| General model description | $DV=f (IDV, P)$ | DV, dependent variable;  F, function; IDV, independent variable; P, parameters |
| --- | --- | --- |
| Structural PK model | 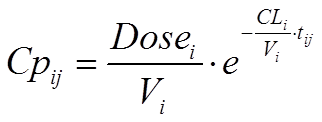 | Cp, plasma concentration; ij, ith concentration in jth subject;  V, volume; CL, clearance; t, time |
| Variability model  (Between subject variability) | 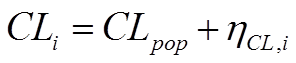 | Fixed effects: CL, clearance; i, individual subject; pop, population  Random effect; η, eta, between subject variability |
| Error model(s)  (residual variability) | 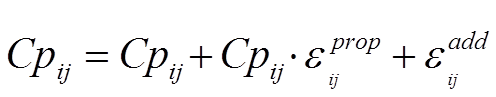 | ε, epsilon, residual variability (or within subject)  prop, proportional; add, additional |
| Covariate models | 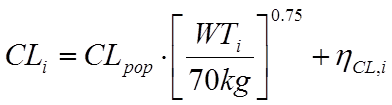 | Allometrically scaled clearance model |
|  | 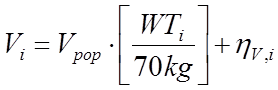 | WT, weight |

## A2C.1 Drug specific safety measures

These measures will depend primarily on the drug´s safety profile. The information on potential toxicity and side effects should be available from animal studies and phases I and II trials in adults and older children. Drug safety can be studied through biomarkers for different organs systems. The biomarkers should only be chosen if relevant to the specific drug to be studied, thus avoiding unnecessary tests and/or blood loss in the neonate. Non-invasive biomarkers should be used whenever possible. A list of suitable biomarkers can be found in

## A2C.2 Drug specific concomitant care and interventions (permitted or prohibited)

Most neonates will have concomitant medication and treatments which should be recorded. Usual intensive care should be permitted as neonates with HIE or meningitis (for example) could have associated multi-organ failure. Ventilated neonates might be sedated and particular attention should be given to potential interaction with this class of drugs.

The study protocol will need to define which drugs are prohibited because of potential negative interaction or adverse effects with the study drug. Alternative drug options should be mentioned in the study protocol so that the neonate can be treated with these alternative drugs as needed.

## A2C.3 Guidance from the EMA and the FDA

To facilitate the development of pediatric formulations, the EMA and the FDA have published guidance documents and detailing formulation aspects in the EMA pediatric investigation plan (PIP) process and the FDA initial pediatric study plan (iPSP) process. In addition, the WHO has also published points to consider:

- Guideline on pharmaceutical development of medicines for pediatric use - EMA/CHMP/QWP/805880/2012 Rev. 2
- List of criteria for screening PIPs with regard to pediatric specific quality issues and referring them to the EMA-PDCO FWG/FDA for discussion
- Pediatric formulation issues identified in Pediatric Investigation Plans.
- FDA Draft Guidance for Industry. Pediatric Study Plans: Content of and Process for Submitting Initial Pediatric Study Plans and Amended Pediatric Study Plans. March 2016.
- Development of pediatric medicines: points to consider in formulation - WHO Expert Committee Working Document 2012.
- ICH E11 CPMP/ICH/2711/99 Clinical Investigation of Medicinal Products in the Paediatric Population
- EMA/CHMP/EWP/147013/2004 Guideline on the role of pharmacokinetics in the development of medicinal products in the paediatric population
- EMA/CHMP/458101/2016 Guideline on the qualification and reporting of 4 physiologically based pharmacokinetic (PBPK) modelling and simulation
- European Commission 2008 Ethical considerations for clinical trials on medicinal products conducted with minors

(ie. blood sampling requirements: 1% total blood volume per sampling, and 4% total BV during 4 weeks)

- EC/93/014 Dose response information to support product authorisation.
- CPMP/EWP/462/95 Clinical investigation of medicinal products in children.
- CPMP/EWP/83561/2005 Guideline on clinical trials in small populations.
- CPMP/EWP/560/95 Note for guidance on the investigation of interactions.
- CPMP/EWP/2330/99 Points to consider on validity and interpretation of meta-analysis, and one pivotal study


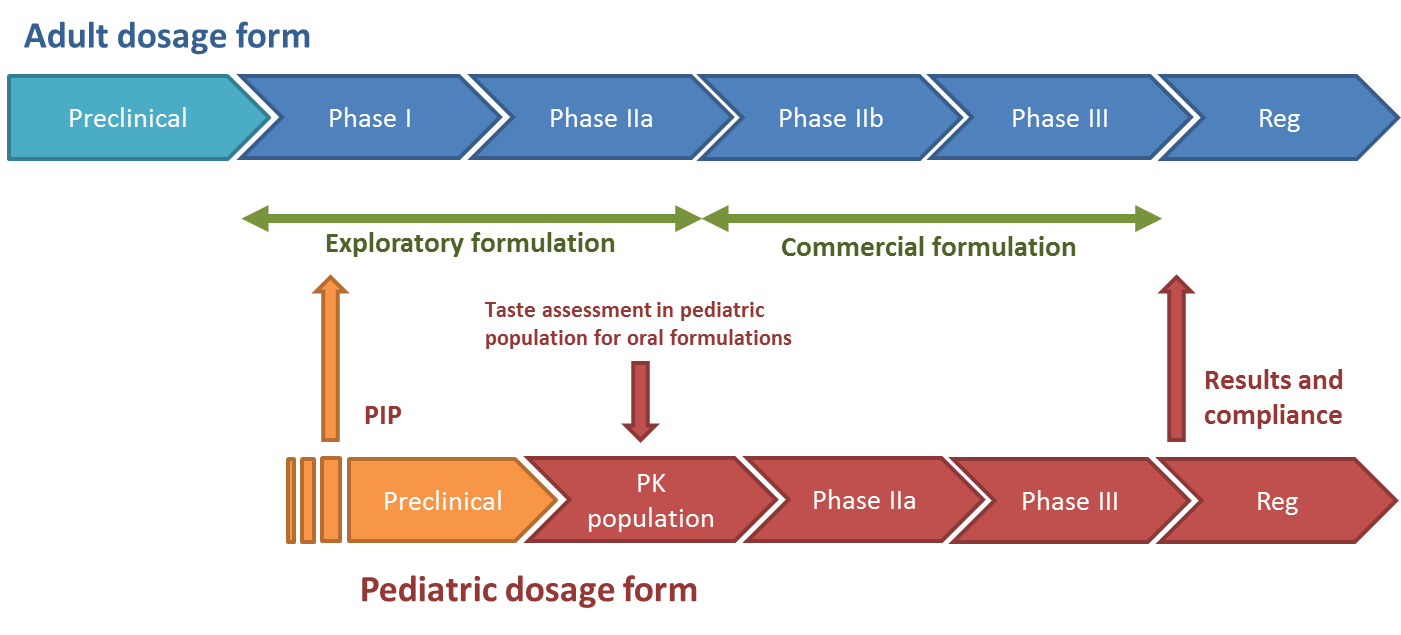


**Figure A2-1: Comparison of timelines for development of adult and pediatric dose formulation**

Appendix 2D List of Suitable Biomarkers in Neonatology

**Biochemical markers by organ system:**

- Metabolic system: blood gas, lactate, blood sugar, ammonia
- Liver: liver function tests, coagulation tests, bilirubin, albumin
- Kidney: urea, electrolytes, creatinine, urine metabolites (might be useful for excretion/PK), urine output (especially neonates with HIE and meningitis)
- Haematology: full blood count (check for suppression of cell lines)
- Other: saliva (for testing drug levels)

**Non-invasive biomarkers:**

- Brain: continuous video-EEG, aEEG, NIRS, cerebral ultrasound, MRI
- Heart: ECG (rhythm check), functional echocardiography, blood pressure, capillary refill time
- Lungs: respiratory rate (apnoea), oxygen saturation measured by pulse oximetry, chest rigidity
- Neurological examination: standardized scheme including muscle tone, reflexes, alertness
- All of the chosen biomarkers will need standardized definitions on how to obtain them and what would be a normal range considered in the study population.

Bibliography

1. Clancy RR, Legido A. The exact ictal and interictal duration of electroencephalographic neonatal seizures. Epilepsia 1987;28(5):537–41.

2. Tsuchida TN, Wusthoff CJ, Shellhaas RA, et al. American clinical neurophysiology society standardized EEG terminology and categorization for the description of continuous EEG monitoring in neonates: report of the American Clinical Neurophysiology Society critical care monitoring committee. J Clin Neurophysiol Off Publ Am Electroencephalogr Soc 2013;30(2):161–73.

3. Stevenson NJ, Boylan GB, Hellström-Westas L, Vanhatalo S. Treatment Trials for Neonatal Seizures: The Effect of Design on Sample Size. PloS One 2016;11(11):e0165693.

4. Lynch NE, Stevenson NJ, Livingstone V, Murphy BP, Rennie JM, Boylan GB. The temporal evolution of electrographic seizure burden in neonatal hypoxic ischemic encephalopathy. Epilepsia 2012;53(3):549–57.

5. Painter MJ, Scher MS, Stein AD, et al. Phenobarbital compared with phenytoin for the treatment of neonatal seizures. N Engl J Med 1999;341(7):485–9.

6. Boylan GB, Rennie JM, Chorley G, et al. Second-line anticonvulsant treatment of neonatal seizures: a video-EEG monitoring study. Neurology 2004;62(3):486–8.

7. van Leuven K, Groenendaal F, Toet MC, et al. Midazolam and amplitude-integrated EEG in asphyxiated full-term neonates. Acta Paediatr Oslo Nor 1992 2004;93(9):1221–7.

8. Castro Conde JR, Hernández Borges AA, Doménech Martínez E, González Campo C, Perera Soler R. Midazolam in neonatal seizures with no response to phenobarbital. Neurology 2005;64(5):876–9.

9. Malingré MM, Van Rooij LGM, Rademaker CMA, et al. Development of an optimal lidocaine infusion strategy for neonatal seizures. Eur J Pediatr 2006;165(9):598–604.

10. Shany E, Benzaqen O, Watemberg N. Comparison of continuous drip of midazolam or lidocaine in the treatment of intractable neonatal seizures. J Child Neurol 2007;22(3):255–9.

11. Abend NS, Gutierrez-Colina AM, Monk HM, Dlugos DJ, Clancy RR. Levetiracetam for treatment of neonatal seizures. J Child Neurol 2011;26(4):465–70.

12. Khan O, Chang E, Cipriani C, Wright C, Crisp E, Kirmani B. Use of intravenous levetiracetam for management of acute seizures in neonates. Pediatr Neurol 2011;44(4):265–9.

13. Sharpe CM, Capparelli EV, Mower A, Farrell MJ, Soldin SJ, Haas RH. A seven-day study of the pharmacokinetics of intravenous levetiracetam in neonates: marked changes in pharmacokinetics occur during the first week of life. Pediatr Res 2012;72(1):43–9.

14. Lundqvist M, Ågren J, Hellström-Westas L, Flink R, Wickström R. Efficacy and safety of lidocaine for treatment of neonatal seizures. Acta Paediatr Oslo Nor 1992 2013;102(9):863–7.

15. Khan O, Cipriani C, Wright C, Crisp E, Kirmani B. Role of intravenous levetiracetam for acute seizure management in preterm neonates. Pediatr Neurol 2013;49(5):340–3.

16. van den Broek MPH, van Straaten HLM, Huitema ADR, et al. Anticonvulsant effectiveness and hemodynamic safety of midazolam in full-term infants treated with hypothermia. Neonatology 2015;107(2):150–6.

17. Pressler RM, Boylan GB, Marlow N, et al. Bumetanide for the treatment of seizures in newborn babies with hypoxic ischaemic encephalopathy (NEMO): an open-label, dose finding, and feasibility phase 1/2 trial. Lancet Neurol 2015;14(5):469–77.

18. Weeke LC, Toet MC, van Rooij LGM, et al. Lidocaine response rate in aEEG-confirmed neonatal seizures: Retrospective study of 413 full-term and preterm infants. Epilepsia 2016;57(2):233–42.

19. Srinivasakumar P, Zempel J, Trivedi S, et al. Treating EEG Seizures in Hypoxic Ischemic Encephalopathy: A Randomized Controlled Trial. Pediatrics 2015;136(5):e1302-1309.

20. Spagnoli C, Seri S, Pavlidis E, Mazzotta S, Pelosi A, Pisani F. Phenobarbital for Neonatal Seizures: Response Rate and Predictors of Refractoriness. Neuropediatrics 2016;47(5):318–26.

21. EMA. Guideline on the Need for Non-clinical testing in Juvenile Animals of Pharmaceuticals for Paediatric Indications. 2008;

22. Davis-Bruno K, de Schaepdrijver L, Annaert P, Campion S, Carleer J, Chapin R, Harrouk W, Laffan SB, McCune S, Moffat GJ, Tassinari MS, Urmaliya V, Chen CL. Nonclinical models of pediatric drug development. 2016;

23. EMEA/CHMP/PEG. Reflection paper: formulations of choice for the paediatric population. European medicines agency pre-authorization evaluation of medicines for human use. London: EMEA; 2006.

24. EMA, CHMP, PDCP. Guideline on pharmaceutical development of medicines for paediatric use [Internet]. 2013;Available from: http://www.ema.europa.eu/docs/en_GB/document_library/Scientific_guideline/2013/07/WC500147002.pdf

25. EMA, CHMP. Guideline on the clinical investigation of the pharmacokinetics of therapeutic proteins [Internet]. European Medicines Agency; 2004. Available from: http://www.ema.europa.eu/docs/en_GB/document_library/Scientific_guideline/2009/09/WC500003029.pdf

26. FDA/CDER/Brian Booth/John KAdavil. Guidance for Industry Bioanalytical Method Validation [Internet]. 2013;Available from: http://www.fda.gov/downloads/drugs/guidancecomplianceregulatoryinformation/guidances/ucm368107.pdf

27. National Cancer Institute, Division of Cancer Prevention. Requirements for: Pharmacokinetic and Biomarker Methods Development [Internet]. 1999;Available from: https://prevention.cancer.gov/sites/default/files/uploads/clinical_trial/biomarkers.pdf

28. Dadgar D, Burnett PE. Issues in evaluation of bioanalytical method selectivity and drug stability. J Pharm Biomed Anal 1995;14(1–2):23–31.

29. Dadgar D, Burnett PE, Choc MG, Gallicano K, Hooper JW. Application issues in bioanalytical method validation, sample analysis and data reporting. J Pharm Biomed Anal 1995;13(2):89–97.

30. FDA, CDER, CBER. Guidance for Industry General Considerations for Pediatric Pharmacokinetic Studies for Drugs and Biological Products DRAFT GUIDANCE [Internet]. 1998;Available from: http://wwwfdagov/downloads/drugs/guidancecomplianceregulatoryinformation/guidances/ucm425885pdf

31. FDA, CDER. General Clinical Pharmacology Considerations for Pediatric Studies for Drugs and Biological Products Guidance for Industry DRAFT GUIDANCE [Internet]. 2014;Available from: http://wwwfdagov/downloads/drugs/guidancecomplianceregulatoryinformation/guidances/ucm425885pdf

32. Knibbe CAJ, Krekels EHJ, Danhof M. Advances in paediatric pharmacokinetics. Expert Opin Drug Metab Toxicol 2011;7(1):1–8.

33. Läer S, Barrett JS, Meibohm B. The in silico child: using simulation to guide pediatric drug development and manage pediatric pharmacotherapy. J Clin Pharmacol 2009;49(8):889–904.

34. Vinks AA. Population Pharmacokinetic–Pharmacodynamic (PK/PD) Modeling of Anti-infective Agents and Its Applications to Individualized Therapy [Internet]. In: Fundamentals of Antimicrobial Pharmacokinetics and Pharmacodynamics. Springer, New York, NY; 2014 [cited 2017 Aug 10]. p. 113–34.Available from: https://link.springer.com/chapter/10.1007/978-0-387-75613-4_6

35. Vinks AA, Emoto C, Fukuda T. Modeling and simulation in pediatric drug therapy: Application of pharmacometrics to define the right dose for children. Clin Pharmacol Ther 2015;98(3):298–308.

36. Anderson BJ, Allegaert K, Holford NHG. Population clinical pharmacology of children: general principles. Eur J Pediatr 2006;165(11):741–6.

37. Anderson BJ, Allegaert K, Holford NHG. Population clinical pharmacology of children: modelling covariate effects. Eur J Pediatr 2006;165(12):819–29.

38. PPRU. Pediatric Pharmacology Research Network (PPRU) Expert Panel Report [Internet]. 2008. Available from: http://www.ppru.org/reports.aspx

39. Beal SL, Sheiner LB. Estimating population kinetics. Crit Rev Biomed Eng 1982;8(3):195–222.

40. Sheiner LB, Rosenberg B, Marathe VV. Estimation of population characteristics of pharmacokinetic parameters from routine clinical data. J Pharmacokinet Biopharm 1977;5(5):445–79.

41. Aarons L. Software for population pharmacokinetics and pharmacodynamics. Clin Pharmacokinet 1999;36(4):255–64.

42. Kiang TKL, Sherwin CMT, Spigarelli MG, Ensom MHH. Fundamentals of Population Pharmacokinetic Modelling : Modelling and Software. Clin Pharmacokinet 2012;51(8):515–25.

43. Broeckman AJ, Sheiner LB, Beal SL. NONMEM Users Guide - Part V. 1994;

44. Ette EI, Williams PJ, editors. Pharmacometrics: the science of quantitative pharmacology. John Wiley & Sons; 2013.

45. Mould DR, Upton RN. Basic concepts in population modeling, simulation, and model-based drug development-part 2: introduction to pharmacokinetic modeling methods. CPT Pharmacomet Syst Pharmacol 2013;2:e38.

46. Ette EI, Williams PJ. Population Pharmacokinetics II: Estimation Methods. Ann Pharmacother 2004;38(11):1907–15.

47. Ette EI, Williams PJ. Population Pharmacokinetics I: Background, Concepts, and Models. Ann Pharmacother 2004;38(10):1702–6.

48. Ette EI, Williams PJ, Lane JR. Population Pharmacokinetics III: Design, Analysis, and Application of Population Pharmacokinetic Studies. Ann Pharmacother 2004;38(12):2136–44.

49. Sherwin CMT, Kiang TKL, Spigarelli MG, Ensom MHH. Fundamentals of Population Pharmacokinetic Modelling. Clin Pharmacokinet 2012;51(9):573–90.

50. Schumitzky A. Nonparametric EM Algorithms for estimating prior distributions. Appl Math Comput 1991;45(2):143–57.

51. Neely M, van Guilder M, Yamada W, Schumitzky A, Jelliffe R. Accurate detection of outliers and subpopulations with Pmetrics, a non-parametric and parametric pharmacometric modeling and simulation package for R. Ther Drug Monit 2012;34(4):467–76.

52. Pharmacometrics Resource at USC Laboratory of Applied Pharmacokinetics (LAPK) [Internet]. 2012;Available from: http://www.lapk.org/

53. Hampson LV, Herold R, Posch M, Saperia J, Whitehead A. Bridging the gap: a review of dose investigations in paediatric investigation plans. Br J Clin Pharmacol 2014;78(4):898–907.

54. Wang S, Zhang J, Lu W. Sample size calculation for the proportional hazards cure model. Stat Med 2012;31(29):3959–71.

55. van der Meer AF, Marcus MAE, Touw DJ, Proost JH, Neef C. Optimal sampling strategy development methodology using maximum a posteriori Bayesian estimation. Ther Drug Monit 2011;33(2):133–46.

56. D’Argenio DZ. Optimal sampling times for pharmacokinetic experiments. J Pharmacokinet Biopharm 1981;9(6):739–56.

57. Vinks AA, Mouton JW, Touw DJ, Heijerman HG, Danhof M, Bakker W. Population pharmacokinetics of ceftazidime in cystic fibrosis patients analyzed by using a nonparametric algorithm and optimal sampling strategy. Antimicrob Agents Chemother 1996;40(5):1091–7.

58. D’Argenio DZ, Schumitzky A, Wang X. ADAPT II user’s guide: pharmacokinetic/pharmacodynamic systems analysis software. 1997:45-52.

59. Duffull S, Waterhouse T, Eccleston J. Some considerations on the design of population pharmacokinetic studies. J Pharmacokinet Pharmacodyn 2005;32(3–4):441–57.

60. Nyberg J, Bazzoli C, Ogungbenro K, et al. Methods and software tools for design evaluation in population pharmacokinetics-pharmacodynamics studies. Br J Clin Pharmacol 2015;79(1):6–17.
